# A Review of the Recent Progress in the Development of Nanocomposites Based on Poly(ether-*block*-amide) Copolymers as Membranes for CO_2_ Separation

**DOI:** 10.3390/polym14010010

**Published:** 2021-12-21

**Authors:** Gabriele Clarizia, Paola Bernardo

**Affiliations:** Institute on Membrane Technology (ITM-CNR), Via P. Bucci 17/C, 87036 Rende, Italy; g.clarizia@itm.cnr.it

**Keywords:** filler, Pebax, CO_2_ separation, mixed-matrix membranes, analysis

## Abstract

An inspiring challenge for membrane scientists is to exceed the current materials’ performance while keeping the intrinsic processability of the polymers. Nanocomposites, as mixed-matrix membranes, represent a practicable response to this strongly felt need, since they combine the superior properties of inorganic fillers with the easy handling of the polymers. In the global strategy of containing the greenhouse effect by pursuing a model of sustainable growth, separations involving CO_2_ are some of the most pressing topics due to their implications in flue gas emission and natural gas upgrading. For this purpose, Pebax copolymers are being actively studied by virtue of a macromolecular structure that comprises specific groups that are capable of interacting with CO_2_, facilitating its transport with respect to other gas species. Interestingly, these copolymers show a high versatility in the incorporation of nanofillers, as proved by the large number of papers describing nanocomposite membranes based on Pebax for the separation of CO_2_. Since the field is advancing fast, this review will focus on the most recent progress (from the last 5 years), in order to provide the most up-to-date overview in this area. The most recent approaches for developing Pebax-based mixed-matrix membranes will be discussed, evidencing the most promising filler materials and analyzing the key-factors and the main aspects that are relevant in terms of achieving the best effectiveness of these multifaceted membranes for the development of innovative devices.

## 1. Introduction

There have been a number of different successful membrane technology applications in industry, particularly in the gas separation field, due to the intrinsic process design simplicity and low capital cost compared to more conventional separation techniques [1]. Polymers occupy a privileged position among the constituent materials of the membranes, mostly due to their easy handling and the ease of preparation by consolidated techniques (e.g., the phase inversion method and dry-jet wet spinning).

However, the trade-off between permeability and selectivity is still a challenge for organic polymers. Nevertheless, other materials (e.g., inorganic materials) present intrinsic features that are even potentially superior to those of the most advanced polymers in terms of operating in more severe conditions, but suffer from important limits related to their brittleness and their high cost for applications on a large scale. A favorable compromise can be achieved by developing nanocomposite systems in which variable amounts of the most innovative and promising fillers are dispersed within a polymeric matrix. In this way, it is possible to enhance the original properties of the neat polymer, without losing its preparation processability [2]. Hybrid membranes obtained by dispersing solid particles within a polymeric matrix are referred to as mixed-matrix membranes (MMMs) and represent an effective strategy for overcoming the trade-off limitations between membrane permeability and selectivity that is an intrinsic feature for polymeric membranes [3].

It should be noted that the simple combination of non-homogeneous phases does not always produce a straight success in terms of performance of the final membrane. In fact, defects at the interface of the phases, non-uniform distribution of fillers within the polymer matrix, and poor stability of heterogeneous systems compared to homogeneous ones are some of the main issues that still to be faced by material science researchers. Indeed, an appropriate design of the filler as well as its compatibility and dispersion into the polymer have a significant effect on the structure and on the separation performance of an MMM. Therefore, it is necessary to select a proper combination of the phases, making the fillers as compatible with the polymer as possible.

The choice of an appropriate filler for a fixed polymer is a crucial challenge in the preparation of effective MMMs for gas separation. Hence, the filler type, which significantly affects the filler/polymer interaction at the interface, governs the gas separation performance. In addition, the design of selective gas transport channels in fillers is a key aspect in order to obtain high-performance MMMs.

Particularly in gas separation, the presence of polar groups in a polymer guarantees a high affinity for CO_2_ with respect to non-polar gases (e.g., CH_4_ and N_2_) making them extremely attractive for carrying out many separations of industrial relevance. The soft poly(ethylene oxide) (PEO) polymer is capable of interacting with CO_2_. However, it would not guarantee the necessary mechanical resistance for membrane applications; for this reason, its combination with a glassy polyamide (PA) block that guarantees mechanical strength, in a block copolymer such as the PEBA, is extremely interesting. Pebax materials combine crystalline and soft regions and a PA/PE ratio that can be adjusted, resulting in several polymer grades.

In the huge category of polymer materials, the rubbery Pebax copolymers are attractive matrices to host a large number of fillers. A comprehensive survey on the effect of nanofillers dispersed in Pebax matrices as membranes was released in 2018 [4]. It examines, in depth, several materials, such as zeolites, metal organic frameworks (MOFs), carbon nanotubes (CNTs), and graphene and graphene oxide (GO) that have been used to prepare MMMs that present improved performance when applied in gas separation. Since then, several studies appeared on the same topic.

The present review embraces the most recent advances, the current status and future prospects of Pebax-based MMMs for gas separation. The main topic addressed in the recent literature is the CO_2_ separation from nitrogen and methane that is of interest to many industrial-scale processes, such as natural gas purification, biogas upgrading, and CO_2_ capture from flue gases, to solve issues of greenhouse gas emission.

The performance of the MMMs will be discussed, grouping the different membranes on the basis of homogenous types of fillers. The new formulations, which are able to enhance the separation properties of the neat polymers, as well as the recent approaches to facing the compatibility issues will be presented (Section 2). The gained insights into the influence of filler type, shape and loading on membrane performance will be discussed on the basis of the instrumental characterization of the developed MMMs (Section 3). The separation performance will be described for the different classes of MMMs, detailing the effects of feed pressure and operating temperature (Section 4).

## 2. Materials for the Analysed MMMs

### 2.1. Polymer Matrix

Table 1 reports the weight fraction of the blocks in the Pebax copolymers for the different polymer grades that are mostly adopted to prepare MMMs for gas separation.

**Figure 1:**
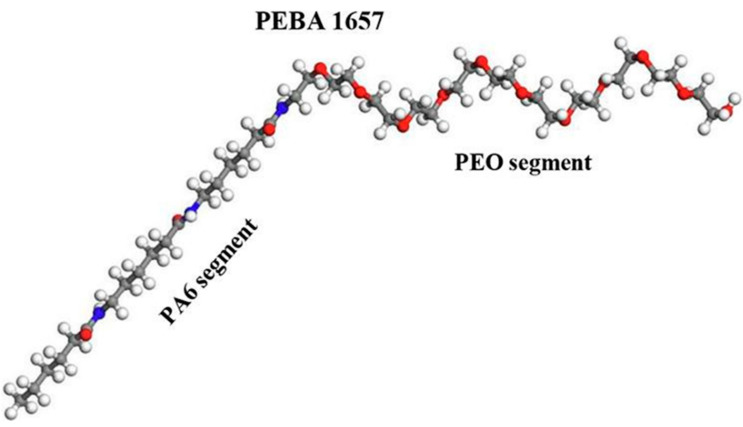
Structure of Pebax 1657. (Adapted from ref. [7]).

The Pebax of 1657 grade is that which is used in the majority of the reviewed studies. Its structure is depicted in Figure 1, which shows the spatial arrangement of EO units with a bond angle of 110° [5]. The repeating units consist of approximately 35 EO units followed by approximately 9 nylon-6 units [6].

Due to the highest content of rigid PA block, Pebax of 1657 grade is characterized by having the lowest gas permeability (as reported in the Section 4). However, this polymer grade displays the highest CO_2_/gas selectivity (as reported in the Section 4). Therefore, it represents a good starting point among the polymeric candidates for CO_2_ separation. On the other hand, Pebax of 2533 grade has been found to be the most permeable to gas molecules owing to its more flexible structure, according to the highest content of the soft PE block.

On the basis of our experience with the preparation of Pebax-based membranes modified by using non-ionic additives as surfactants, Ionic Liquids and filler particles (e.g., MOFs within the EU-funded M_4_CO_2_ project), we can anticipate that Pebax 1657 can be effectively modified by means of blending with appropriate additives or by incorporating suitable fillers.

### 2.2. Filler Materials

**Figure 2:**
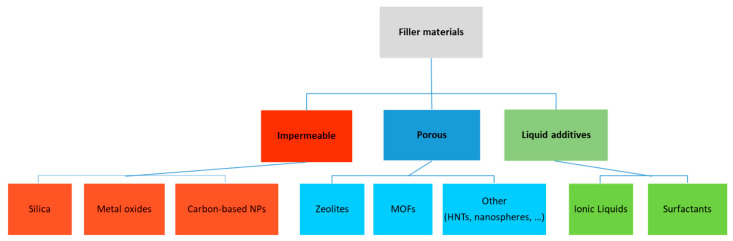
Filler materials adopted for Pebax-based MMMs.

The main classes of materials adopted as fillers within Pebax matrices in the latest studies are reported below. In the majority of the studies, they are nanoparticles. Both porous and non-porous solids were investigated; in addition, liquid additives were also considered to modify the Pebax matrix (Figure 2). In some cases, in order to improve the compatibility between the nanoparticles and the polymer, a compatibilizer was used in the membrane preparation.

#### 2.2.1. Inorganic Impermeable Fillers

An MMM can be seen as a polymeric continuous phase comprising a second dispersed phase in the form of solid particles. Different models are available in order to more realistically illustrate the system, considering even defects or rigid polymeric layers around the fillers [8]. A simple model to describe hybrid membranes (the Maxwell model [9]) would predict a decrease in the permeation flux upon the loading of nonporous fillers in a polymer, since the particles act as obstacles to the permeating molecules, amplifying the tortuosity of the diffusion pathways. However, in some cases, by disrupting the polymer chain packing, impermeable fillers are responsible for an increased permeability for glassy matrices due to an enhanced gas diffusion [10]. In addition, impermeable fillers can provide active sites for sorption and, by increasing the tortuosity for the penetrants, they can enhance the selectivity of the material for certain gas pairs. Impermeable fillers can be functionalized or can offer interlayer spaces, thus providing additional permeation paths and modes.

##### Silica (SiO_2_)

Silica is an inorganic material that is widely used as a filler for nanocomposites due to being inexpensive and combining excellent thermal and mechanical properties [11].

Silica nonporous nanoparticles were dispersed up to 10 wt% [12] and up to 8 wt% [13] to prepare self-supported flat sheet membranes and also for making thin-film composite (TFC) Pebax-based membranes [14].

Other studies exploited the high hydrophilicity of Fumed Silica, due to the abundant silanol groups on its surface, using nanoparticles of 7 nm and 16 nm in size (2–12 wt%) [15], while nanoparticle organic hybrid materials (NOHMs) based on Silica with core/corona/canopy structures and larger sizes (120, 220 and 380 nm) were also investigated [16].

**Figure 3:**
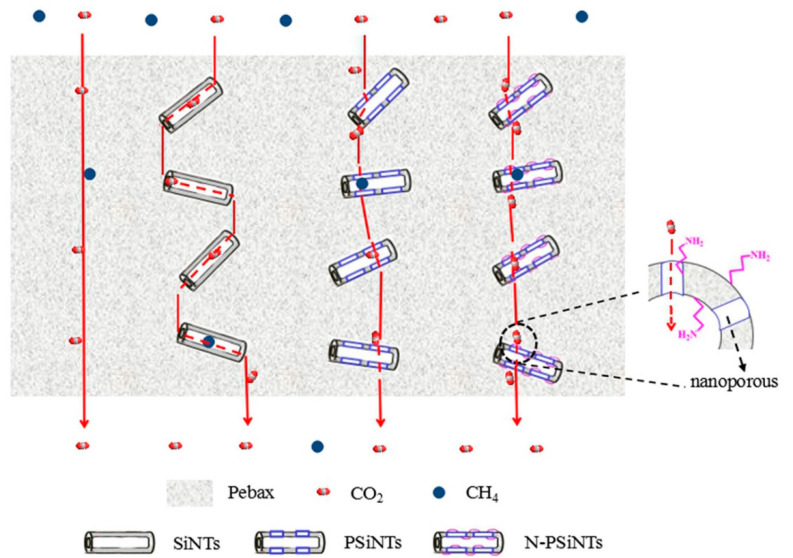
Schematic of the distinct transport modes in MMMs containing non-porous or porous fillers (from ref. [18]).

A comparison of nonporous Silica nanoparticles with porous zeolite and ZIF-8 evidenced the advantage of the last two fillers for the improvement of the gas transport properties of Pebax [17]. As depicted in Figure 3, porous organosilicon nanotubes (PSiNTs) showed distinct advantages in improving the gas permeability compared to non-porous organosilicon nanotubes (SiNTs) [18]. In addition, the amino-modified nanotubes (N-PSiNTs), introduced into Pebax at up to 2 wt% synergistically combined the facilitation of the transport mechanism and the diffusion mechanism [18].

##### Clays

Clays are a wide group of inorganic nonporous layered hydrous silicate structures that are typically used in nanocomposites, mainly for mechanical reinforcement of plastics, as they are abundant in nature and cost-effective [19]. However, while being found to be one of the most prominent fillers in nanocomposite industries, clays did not receive significant attention in the field of gas separation as fillers for mixed-matrix membranes [20]; this was fundamentally because they increase the barrier properties of the original polymer towards permeating gases. Indeed, a 33.3 wt% montmorillonite clay added to Pebax 2533 via the freeze-drying method resulted in aerogels with a 63.5% reduction in oxygen permeability [21]. Similarly, the incorporation of Cloisite 15A, an organically modified montmorillonite, in Pebax 2533 matrix at up to 10 wt%, caused a decrease in CO_2_ permeability as result of the reduction in the adsorption sites of the polymer for gas molecules [22]. Nevertheless, a favorable effect of the increase in feed pressure was observed on CO_2_/CH_4_ selectivity. More promising performances were observed with attapulgite (ATP) dispersed in Pebax 1657 matrix at up to 5 wt% using two different solvents to prepare the MMMs [23]. An increase in CO_2_/N_2_ selectivity was observed as the feed pressure increased, combined with a slight decrease in CO_2_ permeability for all investigated filler concentrations.

Further progress in the development of MMMs was made in the form of composite structures where the ATP/Pebax selective layer was coated on a PAN support with the interposition of a gutter layer, made of extremely permeable material, that allowed the use of a very diluted solution without the risk of infiltration into the pores of the substrate [24]. This approach allowed the increasing of the permeability of CO_2_ by over 40 times compared to the reference ATP-Pebax self-supporting membrane, due to an important reduction in thickness, and, at the same time, a significant increase in selectivity for both the CO_2_/N_2_ and CO_2_/N_2_ pairs.

##### Metal Oxides

Several metal precursors in form of metal oxides are commonly distributed inside polymer matrices as catalysts and reinforcement materials, as well as to modify the transport properties of the original polymer. Their high stability, good availability, and low cost allowed a wide diffusion to produce gas-permeable membranes [25].

Among them, ZnO, in virtue of its chemical stability, biocompatibility, low cost, and toxicity, was used in the form of nanoparticles dispersed in Pebax at 1 wt% [Errore.Ilsegnalibrononèdefinito.], up to 10 wt% [26], and in MMMs based on Pebax/PEG blends [27].

**Figure 4:**
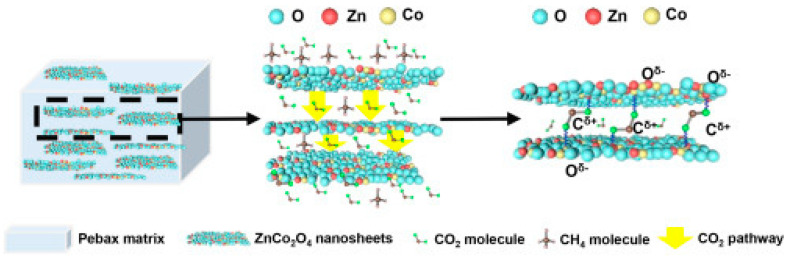
Schematic of the gas transport in MMMs containing 2D porous nanosheets (from ref. [28]).

Two-dimensional porous bimetal oxide zinc cobaltate (ZnCo_2_O_4_) nanosheets were synthesized by a “sacrificial template method” using graphene oxide (GO) as a sacrificial template, resulting in nanosheets with pores of 11.78 nm that provided channels for the transport of gas molecules (Figure 4) [28]. The comparison with ZnO and Co_3_O_4_ nanomaterials shows the synergistic effect of the bi-metal filler in terms of better CO_2_ adsorption and transport properties.

Titanium oxide (TiO_2_) was used to provide unmodified nanoparticles [29] and, after grafting with a silane agent (3-aminopropyl-diethoxymethylsilane), for surface modification with carboxymethyl chitosan [30]. On the other hand, nanoparticles of aluminum oxide (Al_2_O_3_) at up to 8 wt% [31] provided better results in terms of gas separation performance than SiO_2_ and TiO_2_ nanoparticles [13]. In another study, acidic and basic Ionic Liquids (ILs) were used to modify γ-Al_2_O_3_ particles to improve the filler dispersion in Pebax [32]. Finally, Fe_2_O_3_ nanoparticles were successfully dispersed within the polymer matrix (at up to 2 wt% polymer) using a magnetic field of 0.3 T to align the nanofillers [33].

##### Carbon-Based Nanomaterials

Carbon-based nanomaterials in different allotropic forms include carbon nanotubes (CNTs), carbon nanofibers (CNFs), and graphene and graphene-oxide. These carbonaceous materials have received growing consideration owing to a combination of outstanding electrical conductivity, chemical stability, and mechanical stability, as well as reinforcement potential [34].

Single-wall carbon nanotubes (SWCNTs), functionalized with carboxyl groups, were dispersed up to 10 wt% in Pebax^®^ 3000 [35]. Another study investigated the effect of the CNTs loading (2–8 wt%) in blends of Pebax and PEG (up to 50 wt% of PEG), showing a dramatic decrease in CO_2_/CH_4_ selectivity due to membrane plasticizing in mixed gas permeation tests [36].

Multi-walled carbon nanotubes (MWCNTs) were functionalized (MWCNTs-NH_2_) and loaded in Pebax to analyze the key role of the solvent and the temperature of preparation on the filler dispersion [37]. Sedimentation of CNTs was observed in the MMMs prepared with solvents with low molar volume (ethanol/water mixture) that required a long evaporation time, while NMP, a solvent with high molar volume, gelled quickly as the solution cooled down, hindering the mobility of CNTs, and thus, guaranteeing significantly higher transport properties for CO_2_/N_2_ separation.

Nanocomposite membranes were prepared by incorporating covalently grafted polyetheramine (M2070)-carbon nanotube solvent-free hybrid nanofluids (denoted as CNTs NF) that are organic/inorganic hybrids consisting of a structured core grafted with a polymeric canopy [38].

Microporous carbon nanospheres (CNs), prepared with high N contents at different carbonization temperatures, provided accessible inner channels that worked as low-resistance transport pathways for gas molecules [39]. A polyethyleneimine (PEI) layer was used to decorate non-porous Nanodiamonds (ND) on their surface in order to mitigate the agglomeration of the nanofillers, working as both an interfacial binder and a gas-carrier agent [40].

##### Graphene and Graphene Oxide (GO)

Graphene, a two-dimensional (2D) carbon allotrope, has superior thermal, mechanical, and electrical resistance [41]. The 2D structure exposes a large surface area, which is an extremely important requisite for the creation of wider interfaces, resulting in robust composites [34]. Graphene platelets can be exploited as fillers within a membrane to create tortuous paths, while the presence or introduction of structural defects can allow an easy transport of gas molecules. However, graphene clustering occurs within a membrane due to the high charge density of the nanosheets and the lack of polar functional groups.

Low amounts of graphene are able to effectively disrupt the chain-packing arrangement, as also reported for an ultrapermeable polymer (PIM-1), resulting in improved gas permeability [42].

Graphene nanoplatelets (GNPs) loaded in Pebax at concentrations below 1 wt% were used to prepare mixed-matrix composite membranes on PES supports [43]. Graphene was obtained via a green method (jet cavitation-assisted process) and then was N-doped before its incorporation in the polymer matrix at up to 6 wt% using a supermixer [44].

Graphene oxide brings oxygen-containing groups that can interact with the polymeric matrix. In addition, the oxygen-functional groups can be exploited for surface modification of the nanosheets, while the affinity of polar groups as –COOH and –OH on GO sheets with CO_2_ molecules [45] has positive effects on the CO_2_/gas solubility selectivity. On the other hand, the sheeted GO structure can offer molecular sieve channels for gas permeation.

Pristine GO sheets were considered as fillers in Pebax when studying the effect of their dimension on the gas separation performance [46]. By varying the GO lateral sizes (100–200 nm, 1–2 μm, and 5–10 μm), the polymer chains’ mobilities as well as the lengths of the gas channels were effectively manipulated [44]. Graphene oxide (up to 0.3 wt%) was added to blends of Pebax^®^ 1657 and poly(ethylene glycol) (PEG) derivatives [47].

Porous and non-porous GO particles, from 0.02 to 1 wt%, were used to prepare MMMs based on Pebax 2533 [48], showing that porous GO is more effective in increasing the CO_2_ permeability.

Different modifications of GO were also investigated. Imidazole-functionalized graphene oxide (ImGO) nano-sheets [49] and Ionic-Liquid-functionalized graphene oxide (GO-IL) [50] were used as fillers for Pebax^®^ 1657. In particular, the GO-IL was covalently functionalized with 1-(3-aminopropyl)-3-methylimidazolium bromide ionic liquid (IL-NH_2_) that included an amino group with a high affinity with CO_2_, but was also able to enhance the filler–polymer interface compatibility [42]. Other MMMs were developed by incorporating amino-functionalized fillers as aminated-reduced graphene oxide (A-rGO) [51], aminated-partially-reduced graphene oxide (A-prGO) (0–0.6 wt%) [52] and aminosilane-functionalized graphene oxide (f-GO) nanosheets [53].

**Figure 5:**
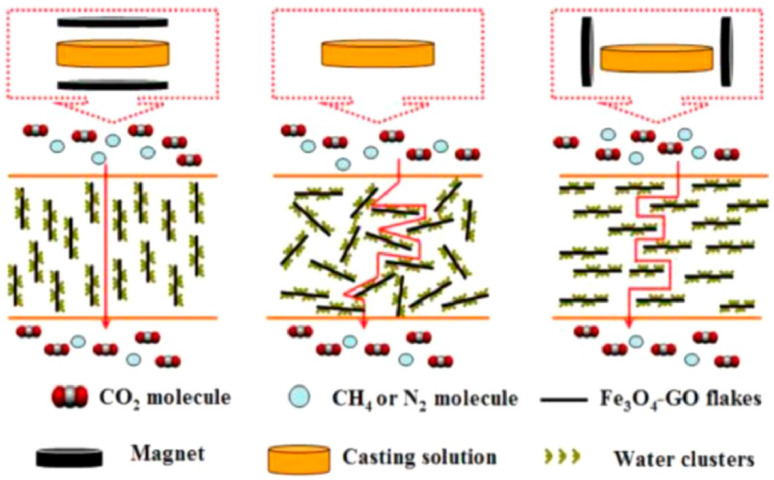
Scheme of MMMs containing flakes with different orientations (from ref. [55]).

GO nanosheets were also modified using polypyrrole and zinc cations [54] or by covalently grafting silane followed by the polyetheramine canopy (M2070) forming solvent-free hybrid nanofluids (denoted as GO NF) [38]. GO flakes functionalized with iron oxide (Fe_3_O_4_–GO) were aligned in the polymer matrix under a magnetic field (Figure 5), demonstrating a better gas separation performance when vertically aligned than MMMs with a random arrangement of Fe_3_O_4_–GO [55].

#### 2.2.2. Porous Fillers

Further advantages with respect to the impermeable fillers are achievable in the case of porous fillers, where additional permeation paths and transport mechanisms are conceivable.

##### Zeolites

Zeolites are inorganic (aluminum silicates) porous crystalline fillers widely employed in fabricating MMMs for gas separation [56].

Pebax-based MMMs were fabricated by incorporating cage-type zeolites at high loadings (NaY, 10–40 wt% [57]; NaY and poly(ethylene glycol), 30 wt% [58]) and –COOH surface-functionalized NaX nanozeolites at low loadings (up to 1.5 wt%) [59]. NaX nano-fillers incorporated into the PEBA polymer (up to 2 wt%) were also used to prepare composite MMMs on a polyethersulfone support, displaying interesting gain in the CO_2_/N_2_ selectivity compared to nonporous SiO_2_ nanofillers [15].

Other studies investigated deca-dodecasil 3 rhombohedral (DD3R) zeolite, a pure silica zeolite with eight-membered ring window openings of 0.36 × 0.44 nm (close to the kinetic diameter of CH_4_ (0.38 nm) and larger than those of CO_2_ (0.33 nm)) at up to 20 wt% [60] and zeolite SAPO-34 with huge cages (1.1 × 0.67 nm) connected to small pore openings (0.38 × 0.38 nm) [61] as fillers. MFI zeolite, with a microporous aperture of ca. 0.55 nm along *b*-axes, was synthetized as nanosheets with thicknesses of 6 nm and, thus, aspect ratios of ca. 200 [62]. Finally, hierarchical Linde Type-T (h-LTT) zeolite could overcome the diffusion limitation via a bimodal porous structure combining micropores and mesopores [63]. It was proposed as a potential filler for Pebax 1657, but the corresponding MMMs were not yet developed [63].

##### Metal–Organic Frameworks (MOFs)

MOFs are crystalline microporous solids obtained by the self-assembly of transition-metal cations and organic linkers. By choosing the appropriate organic ligand, it is possible to tailor their pore size, the chemical functionality of their cavities, and their specific surface area. Therefore, with respect to zeolites, MOFs are more versatile and are widely investigated as fillers.

MOFs were widely considered to prepare Pebax-based MMMs, particularly exploiting Zeolitic Imidazolate Frameworks (ZIFs) that have zeolite-like topologies. ZIFs are porous crystals with a 3-D structure in which tetrahedrally coordinated metal ions (Fe, Cu, Zn, Co) are joined by imidazole linkers. The most common structure, ZIF-8, has large pores of 11.6 Å connected by apertures of 3.4 Å. Larger pore sizes are present in UiO-66 (apertures of 8.0 Å and cavities of 21.0 Å) and in MIL-101(Cr) (apertures of 12–16 Å and cavities of 29–34 Å) [64].

ZIF-8 nanoparticles were prepared in microemulsion with tunable nanosizes (40, 60, 90, and 110 nm) and loaded successfully at a concentration of up to 20% in Pebax [65]. A microemulsion-based mixed linker strategy was developed to introduce amino groups directly during the growth of ZIF-8, resulting in a reduced decline of the surface area (*S*_BET_) of the nanomaterial compared to post-synthetic modification [66]. Other MMMs were prepared by dispersing ZIF-8 in Pebax 2533 with the addition of Pluronic P123 surfactant [67]. A research study focused on Zeolitic Imidazolate Framework cuboid (ZIF-C) nanosheets with tunable thickness from 70 to 170 nm, obtained via a facile method from aqueous polymer solutions, reported better CO_2_ separation performance for the MMMs containing the thickest ZIF-C nanosheets [68].

Another study proposed the use of ZIF-8 hollow nanotubes (HNTs) to introduce high-speed gas transmission channels in MMMs by a three-step method (electrospinning–calcination–hydrothermal), aiming at concurrently improving the gas permeability and selectivity [69].

Hydrophilically modified 2D flakes of the imidazole framework (hZIF-L) improved the membrane performance in CO_2_/CH_4_ separation [70]. The modification induced by tannic acid before filler dispersion within a Pebax 1657 matrix resulted in a double positive effect: the creation of microporous bi-dimensional channels (1.8 nm) and the addition of hydrophilic functional groups on the surface of the ZIF-L flakes. The first result offered accessible gas transport pathways that improved the CO_2_ permeability. The second effectively prevented non-selective interfacial voids and filler agglomeration, conferring a strong binding ability to water and CO_2_ molecules in humid mixed gas tests with enhanced CO_2_/CH_4_ selectivity.

MOF materials (NH_2_-MIL-53(Al), MIL-69(Al), MIL-96(Al), and ZIF-94) with different topologies, chemical functionalities, and pore sizes were employed as fillers in Pebax 1657 at a fixed high loading (25 wt%) [71]. The best performing MOF–polymer composites were prepared by loading MIL-96(Al) as a filler, assuring an increase in CO_2_ permeability and CO_2_/N_2_ permselectivity equal to 25% and 18%, respectively. The highest surface area and CO_2_ uptake of MIL-96(Al) with respect to MIL-69(Al) and ZIF-94 justify this behavior [71]. The beneficial influence on free volume and transport properties of MMMs incorporating MIL-53(Al) with and without NH_2_ functionalization in Pebax or in a blend of Pebax/Cellulose Acetate was proven using molecular dynamics (MD) and Monte Carlo (MC) simulation methods [72].

Nanocomposite membranes containing NH_2_-MIL125 nanofillers, added to the blend of Pebax 1657 and PEG 400, presented a positive effect on the CO_2_/CH_4_ separation performance in comparison with the membranes based on neat Pebax or Pebax/PEG blends [73].

**Figure 6:**
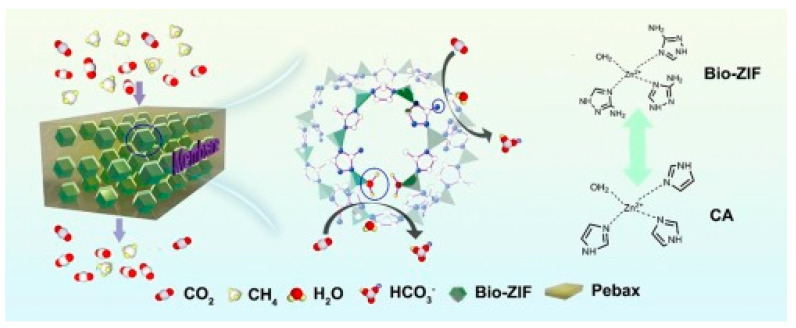
Scheme of MMMs incorporating a bio-inspired filler for CO_2_ separation (from ref. [75]).

Novel MOFs were specifically developed and added to Pebax 1657 as the inexpensive NOTT-300 with high porosity and CO_2_–philic properties [74] and a bio-inspired ZIF (Bio-ZIF) that mimics the structure and function of the carbonic anhydrase enzyme (Figure 6) [75]. The active sites of Bio-ZIF can catalyze the reversible hydration reaction of CO_2_ to bicarbonate, enabling a quick transport of CO_2_ across the MMMs. Interestingly, the Bio-ZIF has a better stability and lower cost than the carbonic anhydrase [75]. A new type of honeycomb-structured UiO-66 MOF was synthesized and amino-functionalized to prepare MMMs [76].

Thin supported MMMs were prepared using MOFs dispersed in Pebax as the selective layer. MOF-801 nanocrystals were incorporated into Pebax on a porous substrate via spinning-coating [77]. ZIF-8, ZIF-67, and CuBTC were dispersed in Pebax at loadings of up to 35 wt% and dip-coated on PAN supports [78]. Other composite MMMs were prepared on polyethersulfone supports with ZIF-8 nano-fillers loaded into the Pebax polymer (up to 2 wt%), displaying interesting gain in the selectivity of CO_2_/N_2_ when compared to silica nanofillers [17]. Nanoparticles of MOFs (ZIF-8, MIL-101(Cr), UiO-66, and ZIF-7/8 core–shells) were dispersed in Pebax^®^ 1657, coating a 2–3 μm thick layer on porous asymmetric polyimide P84^®^ and dense polytrimethylsilylpropyne (PTMSP) supports [62]. UiO-66 MMMs showed the highest permeance for CO_2_, followed by MIL-101(Cr) MMMs, due to their larger pores; ZIF-7/8 MMMs exhibited the lowest permeance according to their narrowest pore distribution; ZIF-8 MMMs combined a good CO_2_/CH_4_ selectivity with a high CO_2_ permeance.

A few studies focused on the development of mixed-matrix thin-film composite using hollow fiber supports that were coated with a thin selective layer of Pebax 2533 incorporating ZIF-8 [79] and amine functionalized UiO-66 nanoparticles via dip-coating [80].

##### Two-Dimensional (2D) Fillers

Two-dimensional titanium carbides, known as MXenes, have attracted growing attention in the membrane research community. MXenes are transition metal carbides, carbonitrides, and nitrides. Starting from MAX phases, where M is an early transition metal and X is carbon and/or nitrogen, MXenes are usually produced by selectively etching the A-element (mainly elements from group IIIA or IVA) [81].

MXene nanosheets were synthesized and loaded at low concentrations (up to 0.3 wt%) into Pebax, and TFC MMMs on PAN supports were produced by spin coating [82]. In another study, a typical MXene, Ti_3_C_2_Tx, was incorporated into a Pebax matrix at up to 20 wt%, resulting in better interfacial interactions compared to GO-filled membranes owing to the rich polar groups present on the MXene surface [83].

MXene in combination with SiO_2_ or HNTs dispersed within a Pebax 1657 matrix at low content (1 wt%) was more effective than alone in enhancing the transport rate of CO_2_ versus N_2_ [84]. Indeed, the 2D MXene nanosheets make additional molecular transport channels available and, at the same time, improve the CO_2_ adsorption capacity, thereby enhancing both the CO_2_ permeance and CO_2_/N_2_ selectivity of Pebax membranes [82].

Layered Double Hydroxides (LDHs), referred to as ‘anionic clays’, are lamellar inorganic solids in which anions are situated in the interlayer galleries and can be ion-exchanged. LDHs are characterized by variable chemical compositions, hydroxyl groups on the surfaces, and interlayer channels, and are considered greener flame retardants [85]. LDHs were decorated with an ionic liquid resulting in [Hmim][NTf_2_]@LDHN nanocages that were added to Pebax exploiting the joint roles of the IL on the external surface, interlamellar spacing, and internal hollow core of LDHNs to improve CO_2_ permselectivity [86]. Exfoliation-free LDH laminates with intrinsic ordered 2D interlayer channels were intercalated with amino acids (Phe with hydrophobic side chains and Glu with hydrophilic side chains) resulting in AA-LDH fillers [87].

Covalent organic frameworks (COFs) were synthesized via a facile sonochemical method as COF-5 nanosheets [88].

#### 2.2.3. Other Solid Fillers

Halloysite nanotubes (HNT) were added at different loadings to a Pebax layer coated on polyetherimide (PEI) supports to produce thin-film composite membranes [89]. In another study, HNTs functionalized with N-β-(aminoethyl)-γ-aminopropyltrimethoxy silane were used [90].

Hollow polypyrrole (PPy) nanospheres with mesoporous shells were synthesized by chemical polymerization using the hard template method [91].

Three anion-pillared hybrid ultramicroporous materials (SIFSIX-2-Cu-i, TIFSIX-2-Cu-I, and GEFSIX-2-Cu-i) were incorporated into a Pebax/PEGDME blend [92].

A series of MMMs based on Pebax 2533 and (core-shell) composite nanoparticles (AAILs@PIM (core-shell) CNPs), with Amino Acid Ionic Liquids (AAILs) in the core and an external dense layer of a polymer of intrinsic microporosity (PIM), were prepared. The AAIL provided high CO_2_ adsorption selectivity, while the coating avoided its loss. Moreover, the PIM shell also provided a good organic interface for the polymer in the MMMs [93].

Copper nanoparticles were incorporated into a glycerol-modified Pebax 1657 (<2 wt%) [94]. Fillers from agricultural wastes (oil palm frond, OPF) were used in Pebax 1657 (2–8 wt%) [95].

#### 2.2.4. Dual Fillers

Dual-fillers are capable of producing synergetic effects in MMMs [96]. Recent studies on Pebax MMMs proposed heterostructured fillers based on Zeolitic Imidazolate Frameworks. ZIF-8 and carboxylated CNTs were compounded to prepare a kebab-like one-dimensional linear composite, ZIF-8@CNTs [97]. Carboxylated CNTs bring many nucleation sites for ZIF-8, thus allowing their growth. In addition, the presence of CNTs in the MMMs resulted in a significant enhancement of the mechanical properties and stability [97]. ZIF-8 particles were in-situ inserted by multiwalled carbon tubes (MWCNTs) (MWCNTs@ZIF-8) [98]. Other heterostructured fillers were obtained through the in-situ growth of ZIF-8 on the LDH surface (ZIF-8@LDH) [99]. The heterostructured fillers resulted in superior gas separation performance with respect to MMMs containing ZIF-8 or LDH as a single filler and with respect to dual fillers (when ZIF-8 and LDH were separately incorporated).

The synergy between 2D nanosheets and a non-2D filler in Pebax MMMs for gas separation was investigated by combining MXene or GO as typical nanosheet in pair with a non-2D filler, SiO_2_, or halloysite nanotubes (HNTs) [84]. Indeed, the dual fillers are more effective in enhancing the separation performance than single fillers. The best combinations are MXene/SiO_2_ and GO/HNTs. The enhanced membrane performance for the former combination depends on the high affinity of the SiO_2_ for CO_2_, whereas for the latter, the presence of HTNs hinders the stacking of GO layers due to a strong steric effect.

#### 2.2.5. Other Additives

Doped Pebax membranes were developed using nonionic polysorbate surfactants that contain many ethylene oxide units and are very CO_2_–philic agents [100,101,102]. The effect of the length of the alkyl chain of nonionic surfactants (Tween20, Tween21, and Tween80) was studied on Pebax 2533-based films in terms of CO_2_ permeability and CO_2_/N_2_ selectivity, which simultaneously increased as the number of N-alkyl groups increased [100]. The addition of two nonionic surfactants (Tween20 and Tween80) to Pebax^®^ 1657 at up to 50 wt% resulted in significant increments in CO_2_ permeability (up two or three times) combined with a substantial maintenance of gas selectivity [100]. A comparison of membranes based on Pebax 2533 or Pebax 1657 doped with polysorbates showed a higher relative increase in permeability using the Pebax of 1657 grade [101]. This was due to the high content of the rigid PA block in the 1657-grade Pebax, which could be plasticized by the surfactants.

Polymer electrolyte membranes were prepared by incorporating anionic surfactants such as Calcium lignosulfonate (CaLS) into Pebax 1657 [103] or KBF_4_ into Pebax 5513 [104]. Aniline molecules were added to the Pebax matrix as semi-mobile carriers for CO_2_ [7].

Different triglycerides (20 wt%) were added to Pebax 2533, exploiting the combined action of the ester group concentration, the plasticization of triglyceride, and the volume resistance of the alkyl chain [105].

## 3. Main Issues in MMMs Development

Apart from the gas permeation tests for the intended separations, which are presented in detail in Section 4, membrane characterization plays an important role in terms of gaining more insight into the particle/polymer interactions and the membrane morphology at a microscopic level, both determining the transport of penetrant molecules. Characterization techniques such as Fourier Transform Infrared Spectroscopy (FT-IR), Differential Scanning Calorimetry (DSC), Thermal Gravimetric Analysis (TGA), mechanical testing, and Scanning Electron Microscopy (SEM) are typically adopted to investigate the compatibility between the nanoparticles and the polymer, the thermal and mechanical stability of the nanocomposite membranes, the polymer chain mobility, and the dispersion of nanofillers into the polymeric matrix. These aspects combined with the permeation properties are fundamental for the successful application of an MMM in gas separation.

In the following, attention will be given to the most important issues of interest for the development of gas separation MMMs, evidencing the means by which the structure–property relationships can be rationally manipulated. Each section will discuss the effects of the filler addition on the polymeric phase as well as on the membrane performance on the basis of the results obtained by the above-mentioned instrumental techniques for the MMM characterization.

### 3.1. Particle/Polymer Interactions

The FT-IR technique gives useful insights into the chemical interactions occurring within MMMs. The addition of some fillers makes it possible to restrict the polymer-chain mobility caused by hydrogen bonding with the copolymer blocks due to specific interactions [60]. On the other hand, the particle/polymer interactions can interrupt inter- and intra-molecular interactions within the Pebax copolymer, extending the amorphous regions (see the “Effect on polymer crystallinity” section).

A partial disruption of the interchain hydrogen bonding between the PA segments was shown by FT-IR spectra on MMMs containing SiO_2_, Al_2_O_3_, or TiO_2_ nanoparticles [13]. Graphene nanoplatelets disrupt the interchain hydrogen bonding of the PA6 segment, resulting in a lowered crystallinity of the PA6 block [43]. The same effect was reported upon the addition of PEGs to Pebax [106].

Some interchain hydrogen bonds are replaced by new interactions between the polymer and the fillers, as reported for Pebax^®^ 1074 membranes incorporating DD3R zeolites [60]. The hydrogen bonding between fillers and the polymeric matrix suggests good interfacial interactions. In the case of ZIF-8 grown in-situ on the LDH surface (ZIF-8@LDH), the presence of ZIF-8 improves the interfacial compatibility between the LDH moiety and the Pebax matrix [99]. An interesting effect of the hydrogen bonding between Pebax chains and GO nanosheets was found, namely a reduced interlayer spacing of GO laminates (to ca. 0.35 nm), which created effective molecular-sieving channels for the CO_2_/N_2_ separation [107]. In the case of functionalized CNTs (CSWCNTs, 10 wt%), the formation of hydrogen bonds between polar carboxyl groups of CSWCNTs and amide/ether groups of Pebax was identified [35]. FT-IR showed favorable interaction of GO with PA rather than PEO in Pebax [47]. Hydrogen bonding interactions between the Ionic Liquid and the amide moieties in Pebax are responsible for the homogeneous dispersion of GO-IL [50]. The IL-functionalized LDHs ([Hmim][NTf_2_]@LDHN) were reported to form hydrogen bonds with both the PA and PEO blocks of the Pebax copolymer, providing an excellent interfacial interaction [86]. The addition of nano ZnO at 0.5 wt% into the Pebax 1657 matrix caused the establishment of ZnO−PEO interactions, while at higher loadings of ZnO, the filler’s self-association (ZnO–ZnO) was found [5].

The occurrence of hydrogen bonding in the MMMs leads to improved mechanical properties (e.g., higher Young’s modulus), as reported for the aniline-loaded membranes [7], for MMMs containing ImGO sheets [49], for Pebax/ZnCo_2_O_4_ [28], and for Pebax/[Hmim][NTf_2_]@LDHN MMMs [86]. Therefore, an increased Young’s modulus is recognized as an indicator of a homogenous dispersion of the fillers in the polymer matrix [86].

### 3.2. Effect on Polymeric Chain Mobility

In general, nanoparticles can affect the polymer’s stiffness and chain mobility, by acting on its *glass transition temperature* (*Tg*), as determined by DSC analysis. Accordingly, they can increase the available fractional free volume (FFV) or decrease it. Other changes observed in the nanocomposites by DSC analysis are related to the melting temperature (*T*m) of the Pebax segments.

The majority of the reviewed studies report a higher glass transition temperature for the MMMs with respect to the neat Pebax. This behavior was observed in MMMs based on ZnO [5], ImGO [49], DD3R zeolite [60], ZIF-8 [65], NOTT-300 [74], GO and MXene [83], [Hmim][NTf_2_]@LDHN [86], AA-LDH [87], AAILs@PIM (core-shell) CNPs [93], NaX, and ZIF-8 [17].

The higher *Tg* for the MMMs can be attributed to a good compatibility between the two phases due to strong interfacial interactions, which tighten the polymer chains at the interface with the nanoparticles [60,86]. The “rigidification” of the polymeric chains around the fillers improves the size-sieving and, thus, the diffusion selectivity is boosted. Typically, a more rigid structure presents better mechanical properties [49].

The shift of *T*g is more pronounced when the nanomaterial loading increases [86,87]. In other cases, as reported for MMMs containing ZnCo_2_O_4_, *T*g initially increases and then decreases with increasing amounts of the fillers, remaining at values higher than the *T*g of the neat polymer [28]. Thus, the filler is effective in restricting the polymer chain mobility.

A more evident increase in *T*g was reported in MMMs containing GO with a larger lateral size [46].

Porous fillers such as NaX and ZIF-8, dispersed into the Pebax 1657, led to a higher *T*g, showing that these nanoparticles were able to restrict the thermal motions of the polymer chains [17].

*T*g also increased upon the incorporation of GO in Pebax/PEG-MEA whereas, in the absence of GO, both *Tg* and *T*m of PEO were decreased with the increasing of the PEG-MEA content in Pebax 1657, indicating a plasticizing effect on the copolymer [47].

A higher melting temperature of the PE phase of the Pebax matrix in MMMs containing CSWCNTs nanofillers at up to 2.1 wt% was related to the rise in rigidity and rearrangement of the polyether phase. At a larger concentration of CSWCNTs (10 wt%), *T*m declined, demonstrating the high flexibility of polyether chains [35]. Instead, lower *T*m values for both PEO and PA chain segments were evidenced for MMMs comprising PIM-HNPs’ hollow nanoparticles due to a chain packing that was modified by the fillers [93].

In a few cases (SiO_2_ [17], γ-Al_2_O_3_ particles modified with ILs [32], CNTs NF, and GO NF [38]), the thermal analysis showed a reduction in *Tg*, indicating the generation of a repulsive interaction and the enhancement of the chain flexibility of Pebax. The decreased *Tg* led to more permeable membranes. The slight decrease in the *T*g observed for SiO_2_-loaded MMMs was attributed to the amorphous nature of the filler [17].

### 3.3. Effect on Polymer Crystallinity

Pebax block copolymers are semi-crystalline and the crystallinity degree of the PEO and PA segments is mainly controlled by the intermolecular bonding (hydrogen bonds) [108]. As also observed in the section “*Particle/polymer interactions*”, the interchain interaction can be disrupted by the fillers that are able to distort the arrangement of the crystalline phases. Therefore, thermal analysis (DSC) as well as X-ray diffraction (XRD) on MMMs typically show a reduced crystallinity for the Pebax segments upon the filler introduction, indicating a transition to a rubberier state. The extension of the amorphous region within the MMMs with less compact chains is connected to an increased FFV of the membranes, which enhances the gas permeability.

In the case of organic-modified CNTs or GO, XRD evidenced a decrease in the crystallinity, in agreement with the decrease in *Tg* found by DSC analysis [38]. The same behavior was reported for MMMs containing SAPO zeolite and it was related to the improved CO_2_/N_2_ selectivity [61]. Additionally, NaY zeolite, increasing from 10% to 40 wt%, determines a reduction in the polymer crystallinity, as evidenced in XRD spectra by the peak at 2*θ* = 24° turning broad with a significantly decreasing intensity [57]. The reduction in the crystallinity degree upon the addition of anion-pillared hybrid ultramicroporous materials to Pebax/PEGDEM was caused by broken interchain hydrogen bonds within the polymer matrix [92]. A reduced crystallinity in the MMMs has been ascribed to the breaking of inter-chain hydrogen bonding between the amide chains in PA blocks [57,90]. 

An increasing PEG-MEA content resulted in a significantly increased crystallinity degree of PEO in Pebax^®^/PEG-MEA blend membranes, demonstrating the incorporation of PEG-MEA in the Pebax^®^1657 matrix [47]. Instead, the crystallinity of PA in Pebax^®^ decreased, leading to improved gas permeability for the blends [45]. Both PEO and PA crystallinity were decreased, as shown by DSC and XRD, upon the loading of GO into the Pebax/PEG-MEA blends [47].

The same behavior was reported for aminosilane-functionalized graphene oxide (f-GO) that decreased the crystallinity and increased the chain mobility of the Pebax matrix [53]. However, the MMMs exhibit a 1.7-times higher Young’s modulus and 1.1-times higher break strength due to the high intrinsic mechanical strength of GO combined with an improved filler dispersion and the semi-interpenetrated Pebax chains in the Si-O-Si network at the interface.

An opposite trend was observed upon the addition of a small amount of GO and MXene, as shown in the XRD spectra for the peak at 2*θ* = 23.8°, which is typical of a PA6 segment that intensifies and becomes sharpened [83]. Thus, these fillers increase the order degree for the PA6 crystalline domains due to interfacial hydrogen bonding resulting in a re-arrangement of the PA6 segments [83].

The use of DMF as a solvent for the membrane preparation, instead of an ethanol/water mixture, causes an increase in the membrane crystallinity and the UiO-66 filler does not change the crystallinity degree, while the NH_2_-UiO-66 filler leads to a more amorphous structure due to the interactions with both the hard and soft segments of Pebax [76].

The crystallinity degree is also affected by the size of the fillers. Larger FS nanoparticles (16 nm) were more effective in decreasing the crystalline regions than smaller FS nanoparticles (7 nm) due to their larger size and higher agglomeration, as shown in SEM analysis [15].

In addition, increasing the filler loading can result in different situations. The crystallinity of the membranes decreased as a result of loading 0.5 wt% of Fe_2_O_3_, while increasing the filler amount from 1 to 2 wt% produced a greater crystallinity degree [33]. DSC analysis demonstrated that an excessive amount of GO may induce further PEO crystallization [83].

Increased amounts of CNTs functionalized with carboxyl groups lowered the crystallinity degree of the PA blocks [35]. Instead, the PE phase of Pebax presented an increased crystallinity upon loading of CSWCNTs at up to 2.1 wt% and a reduction when the filler loading increased at 10 wt%. Therefore, an amount of 10 wt% CSWCNTs, leading to higher flexibility for both Pebax blocks, has a positive effect on CO_2_/CH_4_ permselectivity of MMMs [35].

### 3.4. Effect on Free Volume

The free volume contained within a polymer matrix influences the gas sorption and diffusion [109] and can be altered by the addition of nanoparticles. Experimental evidence was gained using techniques such as Positron Annihilation Lifetime Spectroscopy (PALS) that provides information on the size of the FFV elements, or using XRD, which determines the average chain spacing (d-spacing) of membranes calculated from the Bragg equation. The FFV and density of the membranes can be evaluated according to additive models [47]. Interesting insights into the spatial arrangement of the free volume elements and on their interconnectivity can also be gained by molecular simulation.

The FFV changes depend on the filler loading. Increasing the loading of FS nanoparticles from 0 to 10 wt% led to larger d-spacing of the membrane, thus offering more free volume for the transport of gas molecules [15].

Fe_2_O_3_ nanoparticles produced an increase in the FFV at 0.5 wt% loading, while a greater amount of fillers (up to 2 wt%) reduced the FFV, indicating the blockage of pores and aggregation of nanomaterials, both of which reduced percolation [33].

The incorporation of porous nanoparticles could increase the mean radius of the free volume in MMMs [92]. However, PALS experiments revealed that the free volume of MMMs with 1 wt% GEFSIX-2-Cu-i nanofillers (FFV = 3.63%) was higher than those of MMMs prepared with a higher loading (2.5–10 wt%) of the same filler. This finding is consistent with the overall CO_2_ separation performance decline as GEFSIX-2-Cu-i loading increased from 1 to 10 wt%, due to the severe aggregation of the nanoparticles [92]. An increased inter-segmental gap of the polymer chains was evidenced for MMMs comprising NH_2_-HNTs, resulting in a larger amount of FFV [90]. This behavior is associated with reduced thermal stability.

PALS data confirmed the decrease in FFV as the loading of MXene nanosheets became as high as 5% [83]. In agreement with DSC analysis, the d-spacing decreased with increased *Tg* [83]. The Pebax/[Hmim][NTf_2_]@LDHN MMMs showed smaller d-spacings in comparison with that of pure Pebax membrane (0.397 nm) due to the interaction of the filler with the polymer, as discussed below [85]. The d-spacing was reduced in AA-LDH-loaded membranes, enabling the increase in the CO_2_/CH_4_ diffusion selectivity [87]. Pebax/hZIF-L membranes showed a lower d-spacing compared to the neat Pebax membrane, thus suggesting that the loading of hZIF-L flakes restricts the flexibility of the polymer macromolecules, with beneficial effects on the selectivity for gas mixtures [70].

Larger GO sheets lead to rigid stacking of the polymer chains, reducing the free volume [46]. On the other hand, modified GO-IL display an increase in the interlayer spacing (d-spacing) with respect to pristine GO, as evidenced by XRD [50]. The enlarged d-spacing favors the diffusion of CO_2_ in interlayer channels. The covalent attachment of the IL-NH_2_ to the graphene plane and the ring opening of epoxy groups generate gas passages through the interlayer spacing of modified GO nanosheets [50].

Owing to the abundant hydrophobic groups and low charge density of lignosulfonate ion (LS^2−^), CaLS efficiently disturbs the chain packing of the Pebax 1657 matrix via both metal–organic complexation and hydrophobic interaction, resulting in membranes with tunable FFV [102]. Indeed, the humidity can be used to trigger the polymer swelling with a significant increment in the FFV, but without an extreme enlargement due to the hydrophobic interaction of the CaLS additive with the polymer [103].

The addition of PEG-MEA to Pebax matrix increases the free volume owing to a reduced density for the blends [47]. Although the GO loading results in an increase in the density, the FFV in the MMMs is still high [47].

Some authors investigated the effect of the addition of nanoparticles on the FFV by means of molecular simulation. Modelling of MMMs containing ZnO in the range of 0–1 wt% showed an increased FFV from 0.335 to 0.358 upon the introduction of nanofillers up to 0.5 wt% [5]. Instead, higher loadings caused the FFV to decline owing to stronger ZnO–ZnO interactions that resulted in ZnO clustering [5].

### 3.5. Particle/Penetrant Interactions—Sorption Capacity for Specific Penetrants

Many fillers are characterized by sorption properties (e.g., for CO_2_) that overcome the gas solubility measured in the neat Pebax. These features strongly affect the solubility selectivity term in the solution-diffusion mechanism that governs the gas transport with an evident advantage for adsorptive gas molecules with respect to inert ones.

Zeolites such as MFI have a preferential adsorption affinity, showing a higher saturation capacity for CO_2_ over CH_4_ [62].

An evaluation of the gas solubility showed a higher gas adsorption capacity of Al_2_O_3_ nanoparticles with respect to SiO_2_ and TiO_2_ [13]. The linear CO_2_ sorption isotherm of ZnO and Co_3_O_4_ indicated a weak interaction of the nanomaterial with CO_2_, while the CO_2_ sorption capacity increased for the ZnCo_2_O_4_, to a level that was five times higher than ZnO and 17 times higher than Co_3_O_4_, due to a cooperative effect of the combined metals [28].

A liner sorption isotherm, following Henry’s law, was obtained when analyzing CO_2_ and N_2_ adsorption in rubbery Pebax [77]. Instead, upon the loading of MOF-801 nanocrystals, the amount of gas adsorbed in the MMMs increased sharply for CO_2_ and only slightly for N_2_, indicating that the nanoporous MOF-801 crystals are CO_2_-philic [77]. The combination of ZIF-8 and abundant hydroxyl groups on LDH provide favorable affinity toward CO_2_, significantly enhancing the CO_2_/CH_4_ selectivity [99]. The presence of –COO and –OH groups on the surface of hZIF-L flakes determined a high affinity for CO_2_, increasing its solubility coefficient while the solubility coefficient of CH_4_ was not affected by an increase in the content of hZIF-L flakes [70]. On the other hand, GO also displays a high CO_2_ sorption owing to its polar functional groups, such as –OH, C–O–C and –COOH [52].

Amino groups were inserted in different nanomaterials (e.g., N-PSiNTs [18], NH_2_-ZIF-8 [66], aminated reduced graphene oxide (A-rGO) [51], *A*-prGO [52], aminosilane-functionalized graphene oxide (f-GO) nanosheets [52], and UiO-66-NH_2_ [76,80]) in order to amplify the CO_2_ adsorption on basic sites. The adsorption capacity of amino-functionalized N-PSiNTs was ca. 170% higher than that of PSiNTs (4.72 cm^3^ (STP)/g) [18]. In particular, a significant enhancement in CO_2_ permeability was observed at a filler content of 0.5 wt%, mainly due to the increase in the CO_2_ solubility coefficients, which was in agreement with the CO_2_ adsorption characterization of the nanomaterials. The functionalization of ZIF-8 with amino groups (NH_2_-ZIF-8) resulted in a higher affinity for CO_2_ with respect to the neat ZIF-8, leading to an enhanced solubility selectivity and, thus, to improved separation performances [66]. Furthermore, the insertion mode of the amino groups had an effect on the functionalization efficiency. The introduction of the amino groups during the growth of ZIF-8, according to microemulsion-based mixed linker strategy, led to a minor reduction in the surface area (S_BET_) of ZIF-8, favoring the adsorption of CO_2_ [66]. Indeed, the CO_2_/N_2_ selectivity increased with the increasing of the BET surface area of ZIF-8 [65]. The enhanced permeability was attributed to an improvement in the free volume of the polymer induced by larger sized ZIF-8, while the increased selectivity resulted from the high specific surface area of large sized ZIF-8, which could provide more active sites for CO_2_ capture and great mass transfer resistance for N_2_ [65]. The IL-NH_2_ moieties on the surface of GO nanosheets facilitated the transport of CO_2_ through a reversible reaction [50].

Molecular simulations confirmed the beneficial effect of the amino functionalization in the case of MIL-53(Al) on the CO_2_/CH_4_ separation performance [72].

Some additives were included in the polymer/nanoparticles composites as a third phase in order to provide CO_2_-philic moieties (e.g., EO segments). A polyetheramine canopy (M2070) was used to modify CNTs or GO [38] and silica-based NOHMs [16]. The M2070 imparts a superior CO_2_ selective separation to the MMMs since it brings ethylene oxide (EO) and secondary amine –NH– units that promote the adsorption of CO_2_ by Lewis acid–base interactions and by chemical reaction, respectively. The enhanced CO_2_ selective separation sites for silica-based NOHMs were determined by molecular simulation [16]. An increase in CO_2_ solubility was reported for MMMs comprising both ZIF-8 and Pluronic owing to the joint action of imidazole ligands from ZIF-8 particles and PEO groups from Pluronic P123 [67].

### 3.6. Size Effect

The dimension of the particles has a direct influence on the performance of MMMs. Typically, large size fillers tend to produce more permeable systems, whereas smaller particles are preferred to improve the selectivity of the membrane as a result of their higher surface areas. The dependence of the size is different for porous and non-porous fillers as well as in terms of the function of the nature of the filler.

In general, nanosized fillers are required to prepare membranes for practical applications since thin-film composite membranes present very thin selective layers (ca. 1 micron or less).

Small FS nanoparticles (7 nm) compared to FS-16nm had a more uniform dispersion within the Pebax matrix, leading to a smoother morphology of the nanocomposite membranes, as shown by SEM analysis of the membrane cross-section [15].

Comparing NOHMs particles at the same filler loading, the fillers with a larger core size resulted in a decrease in CO_2_ permeability and N_2_ permeability. At the same filler content, the larger particles were in a lesser amount; thus, the interaction between CO_2_ and EO was less. Indeed, the gas adsorption showed more CO_2_ adsorption capacity in MMMs, including NOHMs with smaller cores [16].

Zeolitic Imidazolate Framework cuboid (ZIF-C) nanosheets with tunable thickness (from 70 to 170 nm) showed the highest CO_2_ permeability in the case of large thickness, while the selectivity was enhanced using the fillers with medium size [68]. The reason for this behavior is ascribed to greater effects of the selective CO_2_ adsorption of thicker ZIF-C nanosheets due to the increased available pathway for gases between layers. On the other hand, the membranes containing thicker 2D nanosheets have lower tortuosity, thus resulting in lower CO_2_/N_2_ selectivity [68].

Size-tunable ZIF-8 nanoparticles (from 40 to 110 nm) produced an enhanced permeability with filler loading and size, whereas the selectivity presented a maximum at a content of 5 wt% [65]. In particular, larger sized ZIF-8 caused an increased permeability due to the larger free volume of the corresponding MMMs, while their high specific surface area increased the selectivity since they provided more active sites for CO_2_ capture and great mass transfer resistance for N_2_.

A GO-PEBA MMM containing 0.1 wt% medium-lateral sized (1–2 μm) GO sheets showed the highest CO_2_ permeation performance compared to small (100–200 nm) and large size (5–10 μm) GO sheets [46].

### 3.7. Shape Effect

A shape effect on the MMMs performance was evidenced, depending on the curvature of the nanofiller surface at the nanoscale [42]. Due to their high aspect ratio, 2D nanosheet materials are expected to strongly interfere with the chain packing of the polymer matrix, resulting in improved gas permeability. A comparison of Graphene with CNTs or spherical silica particles loaded in a superglassy polymer (PIM-1) demonstrated that, on moving from a spherical, to a cylindrical, to a planar filler structure, a marked effect on polymer chain packing can be obtained at extremely low nanofiller amounts [42]. Accordingly, better permeability values are possible with 2D nanofillers at small concentrations (e.g., <0.1 wt%) [42].

Additional paths can be provided within the lamellae [28,50,83]. Indeed, dispersed particles with a bidimensional configuration offer more opportunities to discriminate the gas molecules, taking advantage of an enhanced tortuosity that restricts the diffusion to the larger molecules.

In addition, the orientation of 2D fillers inside the polymer matrix has a fundamental role in the modification of the gas transport, according to the orthogonal or parallel orientation of the sheets with respect to the membrane surface [8].

In the case of GO-IL, the gas molecules can pass through the interlayer spacing of GO nanosheets that are perpendicular to the gas flow; instead, other GO nanosheets positioned in a parallel way to the membrane surface may retard the gas diffusion [50]. This negative effect prevails at high filler loadings of more than 0.05 wt% and decreases the gas permeation [50]. Fe_3_O_4_–GO flakes magnetically aligned in the Pebax matrix provide multiple benefits. The magnetically arranged flakes create shorter transfer paths for gas molecules in the membrane, increasing the CO_2_ permeability. At the same time, the hydroxyl groups on GO surface and the Fe_3_O_4_ improve the CO_2_ solubility selectivity. Furthermore, the better interaction between the GO composites and the polymer reduces interface defects [55].

**Figure 7:**
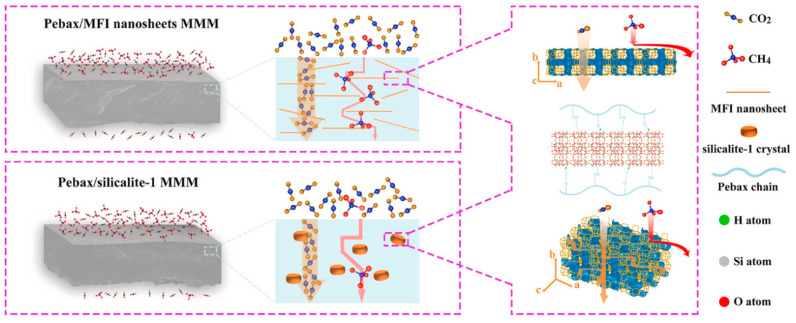
Scheme of MMMs loaded with zeolite as nanosheets or as nanoparticles (from ref. [62]).

The MFI nanosheets, characterized by a high-aspect-ratio (Figure 7), are able to operate as solid and selective barriers in order to allow the preferential passage of CO_2_ molecules through short and straight channels. This behavior leads to a significant increase in both CO_2_ permeability and CO_2_/CH_4_ selectivity, thereby overcoming the trade-off limitation [62].

The 2D-geometry of the nanosheets allows the synergistic action of multiple fillers in combination with respect to a single non-2D filler [84].

The large surface area of ZnCo_2_O_4_ nanosheets [28] or MFI nanosheets [62] results in a good interaction with the Pebax polymer chains and, thus, in defect-free MMMs with enhanced Young’s moduli. The honeycomb-structured UiO-66 MOFs improve the CO_2_ adsorption due to the large surface area [76].

The oriented distribution of ZIF-8 on LDH elevates the CO_2_ concentration around LDH and increases the efficiency of CO_2_-facilitated transport, leading to remarkably enhanced chemical selectivity [99].

The spherical shape and 2-D porosity of MIL-96(Al) have a favorable effect on the transport properties with respect to the platelet MIL-69(Al) with 1-D pore structure [71].

### 3.8. Loading Effect—Strong (Homogeneous Distribution) and Weak (Agglomeration) Points

**Figure 8:**
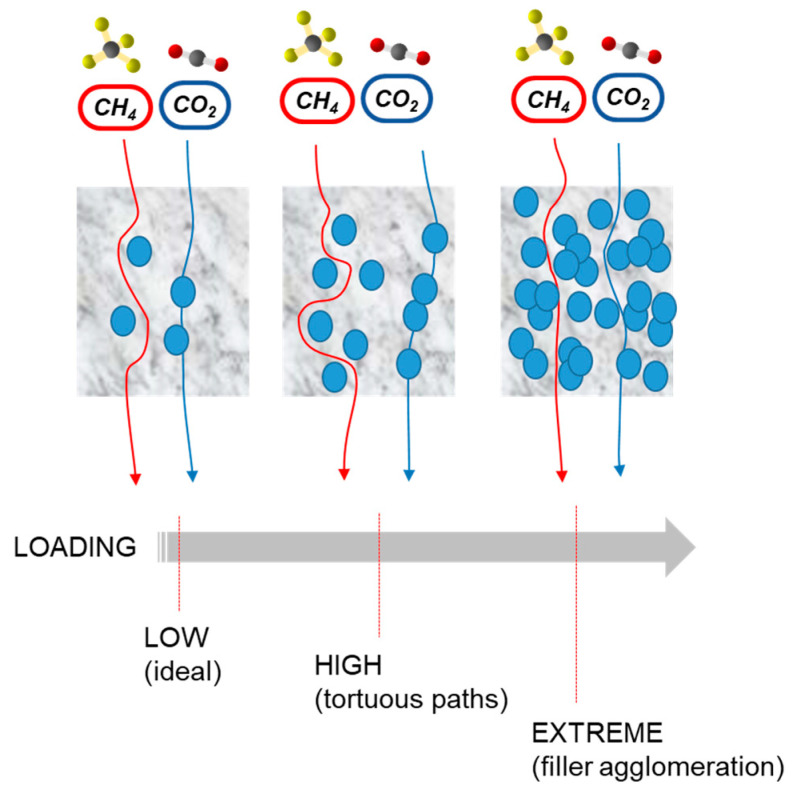
Scheme of the gas transport through MMMs with increasing filler concentration.

Depending on the nature of the filler, it is possible to disperse different amounts in the polymer matrix. Low filler contents could be ineffective in terms of improving the polymer properties at the desired value. However, an increase in the filler concentration over a threshold value has a negative effect, leading to particle agglomeration (Figure 8). Therefore, as a result, MMMs with a high loading can display a reduced permselectivity [14]. Thus, an optimal filler content has to be selected, depending on the filler type. In many cases, only very low contents of the most innovative and expensive fillers are enough to radically modify the polymer characteristics. This aspect is particularly encouraging in view of the application of large-scale separations at competitive costs.

Scanning electron microscopy (SEM) observations, gas permeation measurements, and mechanical testing provide information on the filler dispersion and eventual agglomeration, on the compatibility of the two phases, and on the defect formation within a nanocomposite membrane.

The occurrence of filler stacking or aggregation typically depresses the membrane permselectivity. The threshold value changes with the filler type.

Field emission scanning electron microscopy (FESEM) showed a uniform dispersion in the polymer matrix without significant agglomerations and defects for Silica nanoparticles at up to 10 wt% [12].

After an initial increase in CO_2_/N_2_ selectivity at a low GO loading in Pebax^®^/PEG-MEA blend membranes, a further increase in GO content (>0.3 wt%) resulted in a decrease in both CO_2_ permeability and CO_2_/N_2_ selectivity for the MMMs owing to the combined effect of an enhanced tortuosity of gas diffusion and of GO aggregation [47]. Agglomeration of *A-pr*GO in the polymer matrix was observed when the loading was increased over 0.6%, resulting in an increased permeability of all tested gases but with lower selectivity [52]. The same behavior was reported for bimetal nanosheets at a loading greater than 0.5 wt% [28].

The performance of MMMs with N-doped few-layer graphene (N-FLG) dropped below the upper bound at a concentration of 5% due to agglomeration of the fillers, with a simultaneous decrease in permeability and selectivity [44]. Modelling evidenced that at high concentrations of N-FLG, the CO_2_/N_2_solubility selectivity decreases, since the interaction of FLG sheets with each other results in a saturation effect for the affinity toward CO_2_ [44]. Dual fillers (MXene/SiO_2_ or GO/HTNs) are more effective in improving the gas separation at 1% than at 5% owing to an improved dispersion at low concentrations [84].

At high contents of CNTs NF (>40 wt%), known as the ‘Solvent-free hybrid nanofluids-rich stage’, the CO_2_ permeability of the MMMs sharply increases at the expense of selectivity. The fillers disrupt the arrangement of polymer segments, leading to more open structures, as confirmed by tensile tests evidencing a reduction in the mechanical strength of these MMMs [38].

In the case of thin-film composite membranes, an excessive number of spin-coating cycles (more than three) leads to a reduced permselectivity of the MMMs due to the agglomeration of MOF particles, as evidenced by SEM images [77].

DD3R Zeolite was effectively dispersed at low contents (up to 5 wt%), while at higher contents (10–20 wt%), particles agglomerated and tended to settle, creating an asymmetric membrane [59].

The filler functionalization is an approach that is capable of improving dispersion. Indeed, the surface modification of the fillers improves the compatibility at the interface with the polymeric phase and increases the interlayer spacing, shifting toward higher filler contents with the occurrence of agglomeration [50,90]. GO agglomerated at a loading of 0.2 wt% in a Pebax 1657 matrix, whereas a uniform dispersion was still exhibited by the modified nanomaterial GO-IL at the same concentration [50]. A good dispersion was observed at up to 1.5 wt% for NH_2_-HNTs with respect to the not-modified HNTs, while sedimentation and incipient aggregation of the particles occurred at 2 wt% [90]. SEM micrographs evidenced a good dispersion for the functionalized UiO-66-NH_2_ MOFs, while clear agglomeration was observed for UiO-66 nanoparticles [76]. No agglomerations or defects were observed from the SEM analysis in Pebax MMMs incorporating amino-modified ZIF-8, since the amino groups in ZIF-8 can interact with the amide segment in Pebax, improving the filler–polymer compatibility [66]. However, at loadings exceeding 6 wt% NH_2_-ZIF-8, both the solubility and diffusivity selectivity were reduced due to defect formation [66].

Morphologically homogeneous MMMs, as confirmed by focus ion beam-scanning electron microscopy (FIB-SEM), were obtained by incorporating Nanodiamonds decorated with polyethyleneimine (PEI) as an interfacial binder that develops polymer/filler interfacial interactions, lessening the filler agglomeration [40].

The shape and size of the nanomaterial also play an important role in the agglomeration. The selection of sheeted fillers was found to be capable of containing agglomeration. MXene can be successfully dispersed at much higher concentrations (~20 wt%) than GO (only 5 wt%) in Pebax membranes thanks to the better dispersion and interfacial filler–polymer interaction [83]. In the case of in-situ growth of ZIF-8 on the LDH surface (ZIF-8@LDH), the sheeted LDH suppresses the agglomeration of ZIF-8 at increased loading [99].

GO sheets can lead to poor dispersion and easier formation of stacked structures that penalize the gas transport. This occurrence was found for GO platelets with a larger lateral size [46]. Indeed, for the largest lateral size sheets, the CO_2_ permeability dropped continuously with GO content from 0 to 0.2 wt%, while for small GO-PEBA membranes, it increased with the GO content, and for medium GO-PEBA membranes, the CO_2_ permeability showed a decrease at a GO content of 0.2 wt% [46]. Two-dimensional nanosheets of ZIF-C MOF with smaller thickness tend to stack within the polymer matrix [68].

### 3.9. Mechanical Properties

Table 2 summarizes the mechanical properties, the elastic modulus, the tensile strength, and the elongation at break of some representative MMMs.

Despite a wide scattering of the absolute values, mechanical properties of the neat polymer improve upon the introduction of fillers (higher tensile modulus), but the hybrid materials become less flexible. Thus, the MMMs are typically more rigid, but also more brittle, than the neat polymer. However, the trend does not always continuously and indefinitely increase with the filler content since it is strongly affected by the filler dispersion.

Tensile strength and elongation at break are reduced with an increasing filler loading, while the Young’s modulus is enhanced, indicating that the filler (e.g., ZnCo_2_O_4_ [28]) has a good interaction with the polymer. In some cases, a maximum is observed for the mechanical properties at increasing filler amounts [83]. This behavior is consistent with the maximum found in the selectivity due to the filler agglomeration.

A stronger filler/polymer interaction (e.g., GO-IL/Pebax) leads to moderate increments, with respect to fillers that have a reduced miscibility with the polymer matrix (e.g., GO/Pebax) [50]. Along with the gas barrier improvements, the addition of clay increases the tensile modulus but decreases the tensile strength and elongation at break, probably due to the delamination of the polymer–clay interphase [21].

The humidity also has a depressing effect on the elastic modulus and tensile strength, while it improves the elongation at break [103].

## 4. Gas Separation Performance

### 4.1. Transport Mechanisms Different from Those in Polymers

The commonly accepted “solution-diffusion” mechanism for the permeation of gas species through dense polymeric membranes (P = D × S) [110] can be modified by the presence of the fillers within the matrix. The filler loading can affect the individual permeation terms or both, depending on its properties and on the interplay between the polymeric matric and the nanoparticles. Facilitated transport can occur in the presence of specific additives as a result of preferential adsorption properties or higher affinity of the filler for specific gases (typically CO_2_) and additional paths made available in the matrix. In virtue of this, performance above Robeson’s upper-bound is possible in MMMs.

Different studies compared the effect of porous and non-porous fillers on the MMMs’ performance [17,18,48].

MMMs loaded with non-porous fillers provide transport pathways through the Pebax’s free volume elements and through the spaces at the interface between the Pebax and the fillers.

Additional pathways are made available by porous fillers. However, partial pore blocking can occur in porous fillers when the polymer chains in part penetrate the channels due to a high affinity between the heterogeneous phases. Accordingly, a decrease in the permeation rate is observed with a moderate gain in selectivity if the resulting available spaces are comparable with the kinetic diameters of the gas molecules.

Porous GO was more effective in increasing the CO_2_ permeability than non-porous one dispersed in Pebax 2533 [48]. The same behavior was reported when comparing non-porous SiO_2_ to NaX and ZIF-8 porous nanofillers [17] or for organosilicon nanotubes used as porous or non-porous particles [16].

**Figure 9:**
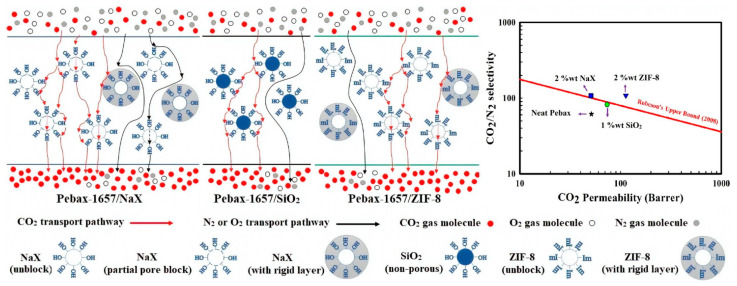
Scheme of MMMs loaded with porous (zeolites and MOFs) and non-porous fillers (SiO_2_) and their separation performance in a Robeson plot (from ref. [17]).

A comparison of nonporous SiO_2_ with porous ZIF-8 and NaX showed a small increase in permeability for the large pore zeolite NaX [17]. Instead, the impermeable SiO_2_ resulted in better permeability values, while the best results were obtained with ZIF-8 fillers. These results were due to a partial blockage of the zeolite pores by the polymer chains due to a good adhesion to the zeolite surface (Figure 9). However, the partially blocked pores of the NaX zeolite acted as molecular sieves and increased the selectivity with respect to neat Pebax or to Pebax/SiO_2_ MMMs.

However, a partial blockage of the porous fillers by the flexible PE segments of the polymer chain can happen, as reported in the case of MMMs containing MOFs (NH_2_-MIL-53) [71] and zeolites (DD3R) [60]. A lower CO_2_ permeability of the MMMs compared to that of the neat polymer was measured for MMMs based on Pebax^®^ 1074 and DD3R zeolite [60]. This behavior depends on a combination of chain rigidification and free-volume reduction in the membranes, and also on the partial pore blockage of the zeolites [60]. A higher loading of DD3R zeolite depresses the permeabilities of CO_2_ and CH_4_, while a low loading (1–5 wt%) the zeolite particles enhances the ideal gas selectivity due to an improved in CO_2_ sorption in the membrane [60].

**Figure 10:**
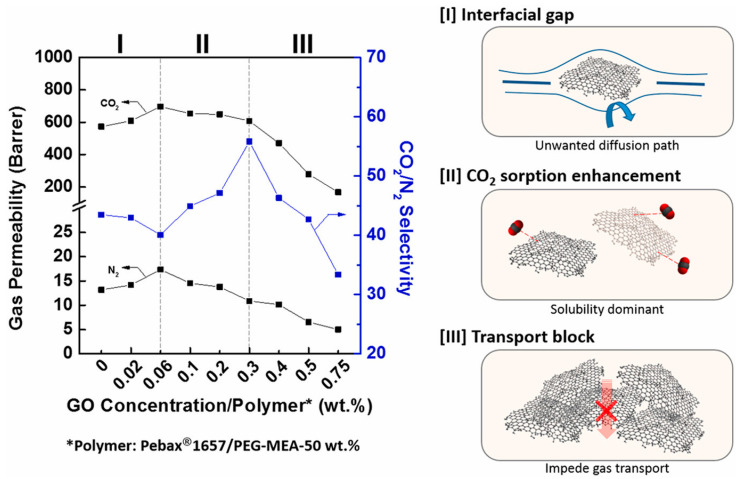
Effect of the GO loading on the permeation properties and schemes evidencing the prevailing transport modes within the MMMs (from ref. [47]).

Two-dimensional-GO loaded in Pebax 1657 blended with PEG-MEA (50 wt%) creates extra diffusional pathways at the interfaces with the polymer causing an increase in CO_2_ permeability. Interlayer channels between few-layered GO can act as molecular sieve. By increasing the GO content, a region with an optimal combination of CO_2_ permeability and CO_2_/N_2_ selectivity was identified in the range 0.06–0.3 wt% (Figure 10) [47].

By analyzing the permeation parameters, it is possible to evidence distinct changes induced by the fillers on the gas diffusivity, on the gas solubility coefficient, or on both permeation terms.

In the case of porous organosilicon nanotubes (PSiNTs), a larger CO_2_ diffusion coefficient was measured in the MMMs [18]. Indeed, BET adsorption–desorption analysis evidenced the presence of micropores on their walls. Accordingly, in addition to one-dimensional channels of SiNTs, the nanotube walls are an extra transport path for gas molecules in Pebax-PSiNTs.

An increase in the CO_2_ solubility coefficients was evidenced for Pebax^®^/PEG-MEA blend membranes incorporating GO nanosheets at up to 0.3 wt%, which resulted in a considerably increased CO_2_/N_2_ selectivity [47]. Indeed, GO has a high affinity for CO_2_ that is enhanced by amino groups [52]. At optimum loading, the amino-functionalized *A*-prGO creates a tortuous pathway that enhances the permeation of CO_2_ in the active layer, but hinders the transport to other gases, increasing the CO_2_ selectivity [52].

ZnCo_2_O_4_ enhances the CO_2_ gas permeability and CO_2_/CH_4_ selectivity since the nanosheets provide pore channels that accelerate the gas diffusion and additional interaction sites for CO_2_ sorption with respect to the neat polymer [28].

A ‘blocking effect’ on CO_2_ was observed for MMMs containing hydrophilically modified 2D imidazole framework flakes (hZIF-L) [70]. The pore structure of these flakes intensifies the diffusion of gas molecules. However, as the filler content increases, the number of functional groups interacting with CO_2_ grows, limiting the diffusion of CO_2_ molecules [70]. Thus, the diffusion selectivity of the MMMs shows an inverse relationship with the hZIF-L amount.

As discussed above, some fillers introduce an additional transport mechanism selectively for CO_2_. For instance, the thickest 2D nanosheets of ZIF-C MOF bring selective adsorption of CO_2_, while for the N_2_, the only mechanism is that of the solution-diffusion model [68]. Instead, the modelling displayed that the addition of N-FLG causes an increase in the diffusivity of N_2_ (three-fold), while that of CO_2_ decreases slightly. This was ascribed to the creation of a tunnel for N_2_ transport by the N-FLG nanolayers, which was confirmed experimentally [44].

Some of the developed MMMs can be categorized as Facilitated Transport membranes since a carrier is used to facilitate the transport of CO_2_. Amino-functionalized porous nanotubes (N-PSiNTs) in MMMs combine the wall porosity that permits a rapid gas transport, thereby intensifying the diffusion mechanism and increasing the CO_2_ permeability, with the reversible reaction between amino groups in the fillers, which provides a facilitated transport mechanism that increases the CO_2_/CH_4_ selectivity [18]. The amino groups on GO in the case of aminosilane-functionalized graphene oxide (f-GO) nanosheets help to construct a facilitated transport pathway along the polymer–filler interface [53]. A similar role was shown by IL-NH_2_ moieties on the GO surface, which are able to react reversibly with CO_2_, and thus, increase the selectivity versus nitrogen and hydrogen as pure gases and in the mixture [50].

Polyethyleneimine (PEI), a solid amine adsorbent for CO_2_ [111], was used to modify Nanodiamonds, thereby functioning as an interfacial binder and as a CO_2_ carrier [40].

The enhanced transport of CO_2_ molecules in Polymer Electrolyte membranes containing KBF_4_ is due the reversible interaction of the dissociated potassium ions with CO_2_ [104]. FT-Raman spectroscopy confirmed the dissociation of potassium ions upon thermal treatment of the material.

The facilitated transport mechanism was added to the solution-diffusion mechanism by introducing an aniline carrier to the Pebax matrix, promoting both the permeability and selectivity of CO_2_. Molecular simulation indicates that aniline molecules can move freely between polymer chains with a high diffusion coefficient, thus enabling the transport of CO_2_ molecules through ‘two hopping and vehicle’ mechanisms. Therefore, compared to the neat membrane, those loaded with aniline show a CO_2_ diffusivity that is increased by 7.5 times [7].

In the case of Ionic Liquid-decorated nanocages, the [Hmim][NTf_2_] on the external surface of LDHN absorbs more CO_2_ molecules than CH_4_, thereby improving the selectivity of CO_2_/CH_4_ [86]. In addition, [Hmim][NTf_2_] in the interlamellar spacing of LDNH dissolves the captured CO_2_ that permeates into LDHN shells. The CO_2_ transport is accelerated since the dissolved CO_2_ combines quickly with CO_3_^2−^ carriers that are present in LDHN. Furthermore, the [Hmim][NTf_2_] within the internal hollow core of the nanocages adsorbs CO_2_. Therefore, the synergy between the IL and the LDHN significantly increases both the permeability and selectivity of CO_2_ [86].

The greatly improved CO_2_ separation performances in the dry state are even better under humidified conditions for MMMs containing aminosilane-functionalized graphene oxide (f-GO) nanosheets [53] and for 2D ZnCo_2_O_4_ nanosheets [28]. Under humid conditions, the interlamellar channels of MXene fillers enable fast and selective CO_2_ transport [83]. In particular, owing to the high upper limit of loading compared to GO, Pebax-MXene membranes show noteworthy performance [83].

In the presence of humidity, water was shown to play a key role in the reversible reaction between CO_2_ and carboxyl/carbonate groups for LDHs that were intercalated with amino acids (AA-LDHs) [87].

The combination of CaLS and water induce a swelling that is not excessive due to the hydrophobic interaction between CaLS and Pebax, resulting in an optimal FFV and moderate salting-out effect that leads to an extremely high CO_2_ permeability (3585 Barrer) and good CO_2_/gas selectivity [103].

In the case of mixed gas testing, competitive sorption phenomena are possible; these can affect the separation performance, as evidenced in a study conducted on ZnCo_2_O_4_ 2D nanosheets [28]. Lower CO_2_ permeability and CO_2_/N_2_ and CO_2_/H_2_ selectivity were measured in mixed gas tests as compared to the corresponding values obtained via pure gas measurements [50].

### 4.2. Defects—Causes and Countermeasures

The filler dispersion within the polymer and the fabrication of membranes with an ultra-thin defect-free selective layer are the key challenges for the development of MMMs. 

A poor adhesion between the heterogeneous phases causes the appearance of some defects at the particle–polymer interface in a composite structure. This aspect is particularly significant in the case of glassy polymers, which have more rigid chains than rubbery polymers.

Defects arise from the creation of void spaces between filler particles and the polymer matrix in the zone surrounding the particle where a rigidification of the polymer chains can happen, depending on the particle/polymer interaction, as discussed above. However, defects also result from the formation of agglomerates. As already discussed, at higher filler concentrations, the probability of defects rises. In addition, a non-uniform filler dispersion negatively influences the membrane performance. The higher density of some particles, with respect to the polymer (e.g., ρ = 3.65 g/cm^3^ for Al_2_O_3_ and ρ = 1.14 g/cm^3^ for Pebax matrix), causes their sedimentation during the casting procedure [32]. Channeling is a consequence of the non-uniform distribution within the host matrix and agglomeration among the particles. This phenomenon is detrimental in terms of the transport properties of the membrane because of the non-selective preferential paths for the gas molecules. As the filler size decreases, the cohesive forces are stronger, favoring this behavior in the presence of high loadings.

Particle pre-treatment (e.g., priming, functionalization, or insertion/grafting of chemical groups) or the addition of plasticizing agents (e.g., Ionic Liquids) are the main approaches to making the heterogeneous phases within an MMM more compatible. Some methods were developed to modify the external surface of the filler particles, and others were applied to make the polymer chains embedding the filler more flexible. The former approach mainly involved depressing the agglomeration tendency of the particles, and the latter involved lowering the rigidity of the host matrix.

Priming and sonication, adopted to incorporate Graphene nanoplatelets (GNPs) in Pebax (at 0.3, 0.5, 0.7 and 1 wt%), were effective in terms of avoiding the agglomerates below 1 wt% [43].

A good dispersion was reported after the functionalization of MOFs (ZIF-8-NH_2_ [66] and UiO-66-NH_2_ [76]) with amino groups since these units can interact with the PA segment in Pebax, thus making the particles more compatible with the polymer. An improved dispersion was also reported for aminosilane-functionalized graphene oxide (f-GO) nanosheets [53].

Grafting with a silane agent (3-aminopropyl-diethoxymethylsilane) and surface modification with carboxymethyl chitosan led to better compatibility between the Titanium oxide nanoparticles and the polymer, better thermal stability of the nanocomposite membranes, and satisfactory dispersion of the nanofillers, as shown via FT-IR, DSC, TGA, and SEM [30].

Poly(ethylene glycols) (PEGs) were used in blends with the Pebax matrix in different works [27,36,47,58,73,92] since they are known to significantly increase the permeability of Pebax [112], while keeping the CO_2_/N_2_ selectivity [47]. A plasticizing effect with a significant reduction in the crystallinity of the PA phase was reported upon the addition of PEG-MEA to Pebax 1657 [45]. Indeed, the addition of low molecular weight PEG to Pebax decreased the activation energy of permeation [113]. The contemporary presence of PEG and nanoparticles can also increase the selectivity. The addition of PEG was found to improve the compatibility of NaY and the membrane matrix, significantly enhancing the CO_2_ permeability and the CO_2_/N_2_ selectivity [58].

Another study proposed Pluronic, a non-ionic block copolymer composed of a hydrophilic group of poly(ethylene oxide) (PEO) and a hydrophobic group of poly(propylene oxide) (PPO), to prepare MMMs based on Pebax^®^ 2533 and ZIF-8 [67]. Glycerol was considered as a low molecular weight additive and was found to provide good CO_2_ affinity [94]. Glycerol alone was effective in enhancing the selectivity but reduced the gas permeability with respect to the neat polymer, while the combination of Glycerol with Cu nanoparticles led to enhanced permeability [94].

Ionic Liquids (ILs) were also exploited as free additives for Pebax [114], resulting in more plasticized matrices with improved permeability. More recently, ILs were introduced in the Pebax MMMs to decorate the fillers before their dispersion within the polymer [50,86,93]. Acidic and basic ILs applied to modify the γ-Al_2_O_3_ particles avoided the sedimentation and agglomeration of the fillers through electrostatic and steric forces [32]. The acidic IL had a better effect on the particle dispersion than the basic IL.

Amino acid ionic liquids (AAILs) were adopted as the core to modify nanoparticles of polymers of intrinsic microporosity (AAILs@PIM (core-shell) CNPs). The AAILs provided high CO_2_ adsorption selectivity, while the PIM shell avoided its loss [93].

In some cases, a dual modification was concurrently performed, as in the work on Pebax/PEG 400/NH_2_-MIL125 nanocomposite membranes in which polyethylene glycol was blended with Pebax, while the filler was amino functionalized [73].

Another approach to solve the problem of nanoparticle agglomeration is the synthesis of hybrid materials such as liquid-like nanoparticle organic hybrid materials (NOHMs) with a polyetheramine canopy [16]. At the same time, Nanodiamonds modified with polyethyleneimine (PEI) had no visible agglomeration, as evidenced by SEM observation even at the highest loading of ND-PEI (1.5 wt%) [40]. The PEI layer on the ND surface successfully improved the interfacial adhesion and dispersion of the NDs in the Pebax matrix [40]. GO flakes functionalized with iron oxide (Fe_3_O_4_–GO) showed reduced interface defects due to a better interaction between the magnetically aligned GO composites and the Pebax matrix [55].

### 4.3. Operation Conditions

Operation conditions (e.g., temperature, pressure) influence the gas transport performance in both single and mixed gases.

Typically, as the temperature increases, the gas permeation rate rises as well, whereas the selectivity trend is the opposite. In fact, the diffusion term increases with temperature for all gas molecules, especially for the less permeable ones, as proven by the activation energy for the permeation process. On the other hand, the solubility term, linked to the condensability of the species, tends to decrease with the temperature, depressing the favorable contribution gained through the most adsorbable species.

MMMs based on ZnCo_2_O_4_ are more sensitive to temperature changes than the pure polymer [28].

A sharp increase in CO_2_ permeability was measured in MMMs based on amino acid ionic liquids encapsulated in a PIM shell, as evidenced by the increase in temperature in the range of 55–85 °C [93]. This was a consequence of a viscosity decay for the CO_2_–AAIL complex induced by the breaking of hydrogen bonds that chemically link CO_2_ molecules to the primary amines of the AAIL. Thus, if in the neat Pebax 2533, the increase in permeability is combined with a decrease in CO_2_/N_2_ selectivity, in the MMMs, it is associated with an increase in CO_2_/N_2_ selectivity, because nitrogen does not experience any further advantage in terms of the reduction in the viscosity with respect to a simple Arrhenius trend [93].

The incorporation of zeolite DD3R into the polymer lowers the activation energy for the permeation of CO_2_, which means that the transport in MMMs is facilitated. On the other hand, a much greater barrier (higher activation energy) to CH_4_ permeation was calculated by adding the zeolite particles [60].

The dependence from the operation pressure is not univocal: a pressure increase causes a denser polymer matrix with a reduction in both gas permeance and selectivity, as found for MMMs incorporating ZnCo_2_O_4_ [28] and for Pebax MMMs with optimal loading of [Hmim][NTf_2_]@LDH (6 wt%) [86]. Nevertheless, the MMM displays a smaller reduction than the neat Pebax membrane [86]. MMMs incorporating PEG and NaY showed a decline for the CO_2_ permeability with the increase in the trans-membrane pressure from 0.10 MPa to 0.25 MPa [58].

In Facilitated Transport membranes, a carrier saturation effect occurs at a high feed pressure, contributing to the deterioration of the CO_2_ separation performance [86,87].

In some cases, CO_2_ molecules can exert a plasticizing action on the polymer matrix when the pressure is raised. Accordingly, the separation selectivity declines, as evidenced in MMMs loaded with AA-LDH fillers for the CO_2_/CH_4_ separation [87].

An opposite behavior was observed for NH_2_-ZIF-8 dispersed at 10 wt% in Pebax 1657, showing an increase in CO_2_ permeability combined with an increase in selectivity moving from 1 to 9 bar [66].

An increase in the permeability of CO_2_ as a polar gas with the increasing of the pressure was observed in MMMs filled with GO-PPy due to the increase in gas sorption [54]. Instead, the permeability of the bulkier CH_4_ molecules was decreased due to the membranes having a more compact structure.

MMMs containing DD3R zeolites displayed a lower relative increase in CO_2_ permeability with the feed pressure than that of the neat Pebax 1074 [60]. This can be ascribed to the decrease in polymer-chain movement caused by the incorporation of zeolite particles. As a further consequence, a suppression of plasticization and swelling of the polymer matrix by CO_2_ was observed following the addition of the zeolite particles.

A positive effect on the permeability of both CO_2_ and CH_4_ was reported in the range of 4–10 bar for NH_2_-HNTs based membranes [90]. However, this increase was larger for CO_2_, thus leading to an increase in the ideal CO_2_/CH_4_ selectivity due to the concomitant effect of a larger CO_2_ sorption capacity for the more condensable species.

Composite MMMs based on MOFs in Pebax 1657, tested for CO_2_/CH_4_ separation at different feed pressures (3–5 bar), showed an improvement in both the CO_2_ permeance and the CO_2_/CH_4_ selectivity with increasing pressure due to the enhanced CO_2_ solubility [60].

Table 3, Table 4, Table 5, Table 6, Table 7, Table 8, Table 9, Table 10, Table 11 and Table 12 report the permeation properties of different MMM types in relation to the CO_2_ separation (CO_2_/N_2_ or CO_2_/CH_4_ gas pairs). However, a direct comparison among several MMMs is quite difficult due to the different testing conditions and Pebax grades. In some cases, since the reported data for neat Pebax differ (see Table 3), the relative percentage change for the permeability or selectivity induced by a filler compared to neat Pebax is reported in round brackets. 

Compared with pure Pebax membrane, the separation performance (permeability and selectivity) was increased following the loading of different fillers. In different studies, the MMMs’ performance exceeded the 2008 Robeson’ upper bound [7,29,36,38,49,51,52,53,60,69,86,88,94].

Enhanced CO_2_ permeability is associated with an increase in filler loading and size, while a trend with a maximum in the curve is characteristic for permselectivity. The data reported for the MMMs in Table 4, Table 5, Table 6, Table 7, Table 8, Table 9, Table 10, Table 11 and Table 12 are those with the optimized filler loading.

Asymmetric and composite membranes are the preferred membrane types in terms of achieving high permeability, reducing the membrane area requirements, and enhancing the productivity of the system. A few papers are devoted to the development of thin-film composite membranes since additional issues have to be addressed, such as the compatibility of the support with the coating solvent and the requirement of very thin selective layers that are defect-free [115]. The data available in literature on the composite membranes, reported in terms of gas permeance, are summarized in Table 12.

## 5. Final Remarks

Based on the main results achieved in the most recent studies, some general considerations on the use of Pebax copolymers as matrices for nanocomposite membranes and on the most promising fillers can be derived.

First of all, the Pebax family showed good compatibility with a large number of fillers based on profoundly different materials.

Most of the research studies focus on the Pebax of 1657 grade, which is less flexible but more mechanically resistant at operating conditions, according to its composition in terms of PE and PA units, as reported in Table 1. The intrinsic transport properties (permeability and selectivity) of the neat polymer are the main reason for this choice by the scientific community.

Several fillers are capable of modifying the structural and gas transport properties of the polymer by virtue of the specific interactions that are established among gas molecules, filler, and host matrix.

A homogeneous dispersion of individual fillers is an essential prerequisite for achieving promising and lasting results: particle type, size, shape and loading are the main factors that influence it. The establishment of new hydrogen bonds between the filler and polymer, which serves as an index of favorable interactions generated between the two phases, modifies the crystallinity of the pristine matrix, typically lowering that of the polyamide fraction, which is non-permeable to the permeation of gases. Thus, an extension of the amorphous region is usually observed.

In any case, the nanoparticles, by disturbing the packing of the polymer chains, lead to a gain of the FV that is available to the passage of the gas molecules. Accordingly, MMMs with a higher loading display an increasing permeability trend. However, agglomeration is usually found at a larger particle loading, resulting in a decay of the permselectivity.

The loading range that leads to noticeable changes in the permeation properties depends on the filler type. Owing to the specific nature of graphene and GO, their content rarely exceeds 1 wt%. CNTs are typically not loaded over 10 wt%. For metal oxide fillers, the content does not exceed 15 wt%, while in the case of MOFs, and even more so for zeolites, loadings of up to 40 wt% are investigated.

Porous fillers are more effective than non-porous fillers in terms of improving the gas transport properties, offering additional pathways to the permeating gas molecules. However, depending on the opening size of their channels, they can experience a partial blockage by the extremely flexible polymer chains. This is also detrimental to the surface properties (e.g., sorption capability) of the nanoparticles.

Fillers with a bi-dimensional aspect are more effective than nanoparticles with a different shape in enhancing the gas permeability, independently of the loading and size.

The orientation of the filler also plays an important role in controlling the transport of gases in the hybrid matrix. Fillers oriented orthogonally to the membrane surface create favorable pathways for the selective permeation of gas molecules, whereas, if they are oriented parallel to the surface, they offer an obstacle to the passage of gases.

Functionalization of the filler is a widely used practice that has a dual purpose: it makes the fillers more compatible with the polymer, improving their dispersion within the matrix and counteracting their agglomeration, but at the same time, it introduces chemical groups that are capable of carrying the selective transport of CO_2_ with respect to other gases.

Particle functionalization is more common for carbon-based materials (e.g., CNTs) than for metal oxides due to higher tendency of the latter fillers to disperse uniformly within the polymer matrix, without generating evident agglomeration or sticking phenomena among nanosheets.

The addition of additives (e.g., Ionic Liquids and PEGs) has been proven effective in terms of making the two heterogeneous phases more compatible.

The combination of different fillers results in better separation properties compared to the use of the individual fillers in MMMs, benefitting from synergic actions in terms of preferential sorption affinity and the presence of selective voids for gas molecules. Equally, bimetal oxides guarantee better performance than single metal oxides at the same loading and size.

An increase in temperature enhances the permeability of all gases through the polymer matrix, according to the preponderant role of the kinetic term (diffusion) over the thermodynamic one (solubility). However, CO_2_ shows a higher adsorption capacity than N_2_ and CH_4_, both in the fillers (especially when they are functionalized) and in the polymeric matrix. This parameter decreases at higher temperatures; therefore, the advantage in terms of permeation rate is lower than that measured for the other gases, resulting in a selectivity decline. The incorporation of the fillers modifies the sensitivity of the hybrid membranes towards temperature, which can be evaluated by means of the activation energy of the permeation for the specific gas. In this case, according to additional transport mechanisms or in the presence of structural changes with temperature, an increase in CO_2_/N_2_ selectivity is possible.

The dependence from the operation pressure is not univocal in terms of either permeation or of selectivity. Indeed, an increase in applied pressure can lead to a denser polymer matrix, but also to a carrier saturation effect with a decrease in both gas permeance and selectivity. However, the opposite trends found in the literature are mainly ascribed to the increase in gas sorption, especially for functionalized fillers.

The humidity strongly affects the gas transport in the mixture with respect to that in the dry state mainly in terms of a higher CO_2_ permeability rather than permselectivity. Indeed, the “swollen” polymer in the wet-state offers a low transport resistance to the gas molecules.

Generally, a reduction in the plasticization and swelling phenomena of the polymer matrix by CO_2_ was observed following the addition of the filler particles, enabling these devices to operate in more severe temperature and pressure conditions.

## 6. Conclusions

Nanocomposite membranes embedding nanoparticles (typically inorganic) in a commercial Pebax matrix have been widely investigated in the last few years as an efficient method to improve the separation properties of current polymeric membranes since they can display the best properties of the combined phases.

The analysis carried out on Pebax-based MMMs incorporating different filler types shows that these membranes were typically tested in view of their use as CO_2_-selective membranes in post-combustion carbon capture applications or for the separation of CO_2_ from methane. On the other hand, the morphology, microstructure, and physicochemical and mechanical properties of the membranes were systematically examined, providing different insights into their structure–property correlations.

Nanofillers with polar properties enhance the permeation of CO_2_ (as a polar gas) versus CH_4_ and N_2_ (as non-polar gases). Several studies focused on introducing additives to improve the compatibility between the heterogeneous phases. Indeed, a poor filler–matrix interaction and the aggregation of filler at a high loading in MMMs restrict their advantage in terms of overcoming the trade-off limitation between permeability and selectivity, as well as weakening the mechanical and thermal properties of the membrane. Additives containing ethylene oxide groups or amine groups promote the mobility of CO_2_ in MMMs. In this regard, the filler functionalization leads to a satisfactory interface compatibility between the polymer chains and the nanoparticles, avoiding the non-selective voids. At the same time, CO_2_-philic functional groups improve the affinity with the CO_2_ molecule, promoting its permeability, solubility, and selectivity with respect to other gases. In the presence of a good adhesion with the polymer, the filler tightens the polymer chains surrounding the particles, as evidenced by the advantageous changes in the crystalline nature and FFV of the MMMs.

An emerging trend is related to the use of two-dimensional (2D) materials as fillers (e.g., graphene oxide) and to the preparation of nanosheets in order to take advantage of additional transport paths. In this respect, 2D fillers represent a benchmark for further investigations.

Porous fillers, when the polymer does not block their cavities, are more effective than non-porous ones, and the combination of different fillers or the incorporation of bimetal oxides promises better performance than the use of a single filler at the same loading and size. In this case, the orientation of the porous filler and its complexity play important roles. When an electrical or a magnetic field can be applied, the aligned fillers lead to better MMMs than membranes with a random distribution.

Overall, the reviewed studies demonstrate that the optimal CO_2_ permeability and selectivity can be attained by combining the structural design of the filler with an optimized loading.

However, further efforts are needed since the majority of the studies focused on self-supported flat-sheet mixed-matrix membranes rather than on the hollow fiber configuration that is the most suited for gas separation. Similar considerations are valid concerning the development of thin-film-supported structures that should assure the highest productivity, but they are still far from the point of tangible wide application in the field of membrane nanocomposites.

## Figures and Tables

**Table 1:** Relative composition of the Pebax grades most used for gas separation.

Pebax Grade	Soft Polyether, wt%	Rigid Polyamide, wt%
1657	40	60
1074	55	45
5513	60	40
2533	80	20

**Table 2:** Mechanical properties of selected Pebax-based MMMs.

Pebax Type	Filler Type	Filler Amount (wt%)	Young’s Modulus (MPa)	Tensile Strength (MPa)	Elongation at Break (%)	Ref.
1657	-	-		0.103	107	[5]
	ZIF-8	5		0.124	134	
	ZIF-8@CNT	5		0.136	214	
1657	-	-	11.49	20.36	983	[28]
	2D zinc cobaltate nanosheets (ZnCo_2_O_4_) with a thickness of about 60 nm	0.5	22.57	15.25	925	
		1	27.39	13.00	779	
		1.5	41.94	12.84	702	
		2	57.88	15.04	757	
		2.5	62.51	13.42	810	
		3	76.35	17.65	893	
1657	-	-		8.47	363	[49]
	Imidazole-functionalized graphene oxide (ImGO) 0.5–1 μm	0.2			387	
		0.5			451	
		0.8		13.53	451	
1657	-	-	201.5	22.6		[50]
	GO	0.2	231.0	14.1		
	GO-IL	0.2	214.7	17.3		
1657	-	-	76.26	35.66	1410	[55]
	Fe_3_O_4_–GO, random alignment		49.26	29.26	1090	
	Fe_3_O_4_–GO, magnetic alignment/vertical	3	48.31	28.89	1060	
	Fe_3_O_4_–GO, magnetic alignment/horizontal		64.2	31.12	1290	
1657	-	-	44	7.4	395	[83]
	GO	1	96	7.5	387	
		2	104	8.9	221	
		5	91	8.2	132	
	MXene	1	105	9.0	386	
		2	185	9.9	376	
		5	155	9.6	382	
		10	114	9.3	389	
		20	97	8.7	391	
1657	-		59.42	18.50	483	[86]
	LDHN	6	52.17	18.21	430	
	[Hmim][NTf_2_]@LDHN	6	92.69	24.89	1262	
1657	-	-	126.4	17.8	863	[91]
	Hollow polypyrrole (PPy) nanospheres	0.5	109.5	12.9	725	
		1	106.2	12.4	661	
		2	102.1	12.1	631	
		7	97.9	11.3	495	
1657	-			4.70	19.2	[98]
	ZIF-8	8		5.70	31.4	
	ZIF-8 particles inserted in situ using multiwalled carbon tubes (MWCNTs@ZIF)	8		8.25	74.2	
1657	-		107 (dry) 80 (humid)	8.4 (dry) 7.0 (humid)	167 (dry) 185 (humid)	[103]
	Pebax–CaLS (60:1)		102 (dry) 61 (humid)	7.6 (dry) 5.3 (humid)	159 (dry) 174 (humid)	
	Pebax–CaLS (30:1)		95 (dry) 57 (humid)	7.2 (dry) 5.0 (humid)	155 (dry) 168 (humid)	
	Pebax–CaLS (15:1)		92 (dry) 60 (humid)	7.6 (dry) 5.3 (humid)	144 (dry) 152 (humid)	
	Pebax–CaLS (7.5:1)		94 (dry) 63 (humid)	7.4 (dry) 5.0 (humid)	129 (dry) 140 (humid)	
2533	-	-		7.7	3.5	[105]
	Triglyceride (TPP)	20		4.7	4.4	
1657	-	-	234.1	40.6	488	[7]
	Aniline molecules	25	379.1	43.3	416	
		50	261.7	28.7	291	
		75	149.2	25.33	344	

**Table 3:** Permeation properties of selected Pebax membranes.

Pebax Type	Additive	Additive Amount (wt%)	Test Conditions	CO_2_ Permeability (Barrer)	CO_2_/N_2_ Selectivity (-)	CO_2_/CH_4_ Selectivity (-)	Ref.
3000	-	-	25 °C, 6 bar	39.7	23.3	13.2	[35]
1657			25 °C, 1 bar	66.5	57.8	19.5	[101]
1657	-	-	25 °C, 10 bar	65.71	81.9		[94]
1657	-	-	30 °C, 2 bar	106	41		[84]
1657	-	-	30 °C, 1 bar Mixed CO_2_/CH_4_ (30/70 vol%)	91 (Dry) 456 (Humid)		17.2 (Dry) 20.9 (Humid)	[99]
1657	-	-	30 °C, 2 bar Mixed CO_2_/CH_4_ (30/70 vol%)	95 (Dry) 470 (Humid)		17.4 (Dry) 20.5 (Humid)	[87]
1657	-	-	30 °C, 2 bar Mixed CO_2_/CH_4_ (30/70 vol%) N_2_ as sweep gas	90 (Dry) 490 (Humid)		17.5 (Dry) 17 (Humid)	[18]
1657	-	-	30 °C, 2 bar (dry) 30 °C, 2 bar (humidified)	89 210	53 38		[83]
1657	-		35 °C, 5 bar	104	38		[97]
1657	-	-	35 °C, 1 bar	83	43		[47]
1657	PEG-MEA	50	35 °C, 1 bar	572	43		[47]
1657	Glycerol (Gl)	15	25 °C, 10 bar	50.42 (−23%)	222.7		[94]
1074	-	-	25 °C, 3 bar	110.67		11.09	[13]
1074	-	-	30 °C, 1.5 bar	145.3 (single) 98.6 (mixed)	19.4 (single) 33.3 (mixed)		[58]
1074	-	-	30 °C, 1.5 bar	145.3	19.2		[61]
2533	-	-	35 °C, 1 bar	364.61	23.80		[48]
2533	-	-	35 °C, 5 bar	298	24		[105]

**Table 4:** Permeation properties of selected Pebax-based MMMs filled with inorganic (typically non-porous) particles.

0	Filler Type	Filler Amount (wt%)	Test Conditions	CO_2_ (Barrer ^1^)	Selectivity (-)	Ref.
1074	SiO_2_ nanoparticles	10	25 °C, 3 bar	105.94	26.09 (CO_2_/CH_4_)	[12]
1074	SiO_2_ nanoparticles (particle size 20 nm)	8	25 °C, 3 bar	152.10	13.28 (CO_2_/CH_4_)	[13]
1657	Non-porous SiO_2_	1	25 °C, 4 bar	73.65 (+44%)	81.82	[17]
1657	Fumed silica (FS) (7 nm)	10	25 °C, 12 bar	72.91	113.92 (CO_2_/N_2_) 28.04 (CO_2_/CH_4_)	[15]
1657	Silica nanoparticle organic hybrid materials (NOHMs) Liquid-like nanoparticle (120 nm) Canopy: polyetheramine M2070 (P-NOHMs-120-(15))	15	25 °C, 2 bar dry feed gas	246.7	66.4 (CO_2_/N_2_)	[16]
1657	Non-porous organosilicon nanotubes (SiNTs)	0.5	30 °C, 2 bar Mixed CO_2_/CH_4_ (30/70 vol%) N_2_ as sweep gas	130 (Dry) 750 (Humid)	21 (Dry) 24 (Humid) (CO_2_/CH_4_)	[18]
	Porous organosilicon nanotubes (PSiNTs)	0.5		150 (Dry) 900 (Humid)	23 (Dry) 25 (Humid) (CO_2_/CH_4_)	
	Porous organosilicon nanotubes amino-modified (N-PSiNTs)	0.5		160 (Dry) 972 (Humid)	24 (Dry) 29.2 (Humid) (CO_2_/CH_4_)	
1657	ZnO nanoparticles	0.5	25 °C, 14 bar	140 (sim. 132.29)	95 (sim. 96.56) (CO_2_/N_2_) 30 (sim. 29.13) (CO_2_/CH_4_)	[5]
1657	ZnO nanoparticles	10.0	30 °C, 3 bar	149.8 (+13%)	24 (CO_2_/CH_4_) (+21%)	[26]
1657/PEG400 (40 wt%)	ZnO	4	25 °C, 7 bar	94.49	31.58 (CO_2_/CH_4_)	[27]
1657	2D nanosheet zinc cobaltate (ZnCo_2_O_4_) (thickness of about 60 nm)	0.5	25 °C, 2 bar Mixed CO_2_/CH_4_ (10/90 vol%)	139.10 pure (+165.67%) 118.6 mixed	15.38 pure (CO_2_/CH_4_) (+75.57%) 32.46 mixed	[28]
1657	2D nanosheet zinc cobaltate (ZnCo_2_O_4_)	1	Mixed gas, Wet	415.96	31.29 (CO_2_/CH_4_)	
1074	TiO_2_ nanoparticles (21 nm)	8	25 °C, 3 bar	150.31	13.18 (CO_2_/CH_4_)	[13]
1657	TiO_2_	8	30 °C, 3 bar	172.32	24.79 (CO_2_/CH_4_)	[29]
1657	TiO_2_ modified by silane grafting (AS-TiO_2_)	3	25 °C, 20 bar	188.6	84.9 (CO_2_/N_2_)	[30]
	TiO_2_ modified by grafting with carboxymethyl chitosan (CMC-TiO_2_)	3	25 °C, 20 bar	194.6	82.4 (CO_2_/N_2_)	
1657	Al_2_O_3_	8	25 °C, 3 bar	159.27	24.73 (CO_2_/CH_4_)	[31]
1074	γ-Al_2_O_3_ nanoparticles (20 nm)	8	25 °C, 3 bar	163.87	14.24 (CO_2_/CH_4_)	[13]
1657	γ-Al_2_O_3_/ILs acidic IL-modified particles (0.5 µm)	10	25 °C, 7 bar	126 (+47%)	101 (CO_2_/N_2_) (+ 124%)	[32]
	γ-Al_2_O_3_/ILs basic IL-modified particles (0.5 µm)	10	25 °C, 7 bar	108	78 (CO_2_/N_2_)	
1657	Fe_2_O_3_ magnetic	1.5	14 bar	165.6	157.25 (CO_2_/N_2_) 55.95 (CO_2_/CH_4_)	[33]

^1^ Barrer = 10^−10^ cm^3^ (STP) cm cm^−2^ s^−1^ cmHg^−1^.

**Table 5:** Permeation properties of selected Pebax-based MMMs filled with carbon materials.

Pebax Type	Filler Type	Filler Amount (wt%)	Test Conditions	CO_2_ (Barrer ^1^)	Selectivity (-)	Ref.
3000	Carboxyl-functionalized single-wall carbon nanotubes (CSWCNTs) (length 30 nm; outer d 1–2 nm; inner d 0.8–1.6 nm)	10	25 °C, 6 bar	53.2	106.4 (CO_2_/N_2_) 31.3 (CO_2_/CH_4_)	[35]
1657/PEG200	CNT	8 CNT 50 PEG	25 °C, 14 bar mixed CO_2_/CH_4_ (50/50 vol%) 25 °C, 12 bar 40 °C, 12 bar	302 (pure) 138 (mixed) 193 (mixed)	45 CO_2_/CH_4_ (pure) 19 CO_2_/CH_4_ mixed 15.7 CO_2_/CH_4_ mixed	[36]
1657	MWCNT-NH_2_ (outer diameter 8–15 nm, length ∼50 µm, 0.45 wt% NH_2_) NMP as solvent	6	30 °C, 3.5 bar	174	32 (CO_2_/N_2_)	[37]
		6	45 °C, 3.5 bar	285	57 (CO_2_/N_2_)	
		6	60 °C, 3.5 bar	405	51 (CO_2_/N_2_)	
1657	Carbon nanospheres (CNs-600) (650 nm)	0.5	25 °C, 4 bar mixed CO_2_/N_2_ (10/90 vol%)	100 (pure gas) 97 (mixed gas)	76 (pure gas) 64 (mixed gas)	[39]
1657	Nanodiamonds (ND) 5–10 nm	0.5	35 °C, 2 bar feed pressure and 0.015 bar downstream	46	35.5	[40]
	Nanodiamonds (ND) decorated with polyethyleneimine (PEI) (5–10 nm)	0.5	35 °C, 2 bar feed pressure and 0.015 bar downstream	50	51	
1657 on PES support	Graphene nanoplatelets (GNP)	0.7	25 °C and 4 bar	45 (+68%)	112 CO_2_/N_2_ (+50%), 9.9 O_2_/N_2_ (+28%)	[43]
1657 on PVDF support	N-doped few-layer graphene (N-FLG) (thickness ~4 nm, ~10 layers)	4	Room T, 1–2 bar	239.8	95.5 (CO_2_/N_2_)	[44]
1657/ PEG-MEA	Graphene oxide (GO)	0.3 GO 50 PEG-MEA	35 °C, 1 bar	600	55.8 (CO_2_/N_2_)	[47]
1657	GO sheets Medium-lateral sized (GO-M) (1–2 μm, d-spacing of 0.8 nm)	0.1	single: 25 °C, 3 bar mixed: 25 °C, 1 bar CO_2_/N_2_ (50/50 vol%)	95 Single gas 75 Mixed dry 110 Mixed humid	85 Single gas (CO_2_/N_2_) 72 Mixed dry 80 Mixed humid	[46]
1657	GO (sheet size: 500–1000 nm, thickness: 1.5–2.0 nm)	1	30 °C, 2 bar (dry)	110	67 (CO_2_/N_2_)	[83]
		10	30 °C, 2 bar (humidified)	420	64 (CO_2_/N_2_)	
1657	Imidazole-functionalized graphene oxide (ImGO) (0.5–1 μm)	0.8	25 °C, 8 bar	76.2	105.5 (CO_2_/N_2_) (+46.0%)	[49]
1657 on PVDF support	Ionic-Liquid-functionalized graphene oxide (GO-IL)	0.2	25 °C, 4 bar	143	79.4 (CO_2_/N_2_) 13.8 (CO_2_/H_2_)	[50]
1657 on PSf support	Aminated partially reduced GO nanofiller (*A*-prGO)	0.1	Room T, 4 bar	47.5	105.6 (CO_2_/N_2_) 23.75 (CO_2_/CH_4_)	[52]
1657	Aminosilane-functionalized GO Nanosheets (f-GO)	0.9	35 °C, 2 bar, humidified mixed CO_2_/N_2_ (20/80 vol%) mixed CO_2_/CH_4_ (30/70 vol%)	934.3	71.1 (CO_2_/N_2_) 40.9 (CO_2_/CH_4_)	[53]
1657	GO nanosheets	0.1	25 °C	107 (4 bar) 117 (10 bar)	104 (4 bar), 77 (10 bar) (CO_2_/N_2_) 22 (4 bar), 26 (10 bar) CO_2_/CH_4_	[54]
1657	GO nanosheets modified by polypyrrole (GO-PPy) (100 to 200 nm)	0.1	25 °C	100 (4 bar) 122 (10 bar)	107 (4 bar), 123 (10 bar) CO_2_/N_2_ (+62%) 22.5 (4 bar), 30 (10 bar) CO_2_/CH_4_ (+51%)	[54]
1657	GO nanosheets modified by polypyrrole and zinc cations (GO-PPy-Zn)	0.1	25 °C	118 (4 bar) 131 (10 bar)	83 (4 bar), 119 (10 bar) CO_2_/N_2_ (+58%) 24 (4 bar), 30 (10 bar) CO_2_/CH_4_ (+56%)	[54]
1657	Covalently grafted polyetheramine (M2070)-carbon nanotube solvent-free hybrid nanofluids (CNTs NF)	30	25 °C, 1.0 bar Dry mixed CO_2_/N_2_ (20/80 vol%)	225 (pure 2 bar) 180 (mixed 2 bar) 332 (mixed 1 bar) (+442%)	61 (pure 2 bar) 60 (mixed 2 bar) 72 (mixed 1 bar) (+77%) CO_2_/N_2_	[38]
	Covalently grafted polyetheramine (M2070)-graphene oxide solvent-free hybrid nanofluids (GO NF)	15	25 °C, 1.0 bar Dry mixed CO_2_/N_2_ (20/80 vol%)	150 (pure 2 bar) 140 (mixed 2 bar) 248 (mixed 1 bar)	52 (pure 2 bar) 48 (mixed 2 bar) 56 (mixed 1 bar) CO_2_/N_2_	
1657	Graphite oxide flakes functionalized with iron oxide (Fe_3_O_4_–GO) magnetic alignment/vertical	3	25 °C, 2 bar Mixed gas CO_2_/CH_4_ or CO_2_/N_2_ (10/90 vol%)	538	75 (CO_2_/N_2_) 47 (CO_2_/CH_4_)	[55]
2533	GO	0.02	35 °C, 1 bar	371.39	24.00 (CO_2_/N_2_)	[48]
	Porous (PGO)	0.02	35 °C, 1 bar	397.35	23.75 (CO_2_/N_2_)	
	Polyetheramine-functionalized graphene oxide (PEAGO)	0.02	35 °C, 1 bar	380.44	24.19 (CO_2_/N_2_)	

^1^ Barrer = 10^−10^ cm^3^ (STP) cm cm^−2^ s^−1^ cmHg^−1^.

**Table 6:** Permeation properties of selected Pebax-based MMMs filled with zeolites.

Pebax Type	Filler Type	Filler Amount (wt%)	Test Conditions	CO_2_ (Barrer ^1^)	Selectivity (-)	Ref.
1657	MFI nanosheets	5	25 °C, 2 bar	188.9 (+63.5%)	29.9 (CO_2_/CH_4_) (+76.4%)	[62]
		5	25 °C, 2 bar Mixed CO_2_/CH_4_ (50/50 vol%)	159.1 (+63.5%)	27.4 (CO_2_/CH_4_) (+76.4%)	
1657	NaX (mean particle size 55 nm)	2	25 °C, 4 bar	50.70	from 61.53 to 107.13 (CO_2_/N_2_) 6.06 (O_2_/N_2_)	[17]
1657	NaY	40	30 °C, 2 bar	131.8	130.8 (CO_2_/N_2_)	[57]
1657 on PES support	NaX (40–90 nm)	1.5	25 °C, 6 bar	95	100 (CO_2_/N_2_), 32 (CO_2_/CH_4_) 3 (N_2_/CH_4_)	[59]
	NaX-COOH (40–90 nm)	1.5	25 °C, 6 bar	187.76	288.86 (CO_2_/N_2_), 57.41 (CO_2_/CH_4_) 5.03 (N_2_/CH_4_)	
1074/PEG	NaY (1.7 μm)	30 NaY and 20 PEG	30 °C, 1.5 bar Mixed CO_2_/N_2_ (15/85 vol%)	172.6 (single) 140.1 (mixed)	107.9 (single) 166.7 (mixed) CO_2_/N_2_	[58]
1074	DD3R (ca. 10 μm)	5	30 °C, 5 barg	120	31 (CO_2_/CH_4_)	[60]
1074	SAPO (0.45 μm)	5	30 °C, 1.5 bar	98.2	72.0 (CO_2_/N_2_)	[61]

^1^ Barrer = 10^−10^ cm^3^ (STP) cm cm^−2^ s^−1^ cmHg^−1.^

**Table 7:** Permeation properties of selected Pebax-based MMMs filled with MOFs.

Pebax Type	Filler Type	Filler Amount (wt%)	Test Conditions	CO_2_ (Barrer ^1^)	Selectivity (-)	Ref.
1657 on PAN support	CuBTC (range size distribution 60–500 nm)	35	25 °C, 12 bar Mixed CO_2_/CH_4_ (10/90 vol%)	94.4	18.84 (CO_2_/CH_4_)	[78]
	ZIF-67 (mean size around 400 nm)	15	25 °C, 12 bar Mixed CO_2_/CH_4_ (10/90 vol%)	42.2	17.36 (CO_2_/CH_4_)	
	ZIF-8 (mean size < 100 nm)	35	25 °C, 12 bar Mixed CO_2_/CH_4_ (10/90 vol%)	94.4	20.35 (CO_2_/CH_4_)	
1657	ZIF-8 (90 nm)	5	20 °C, 1 bar	99.7 (+25%)	59.6 (CO_2_/N_2_) (+25%)	[65]
1657	ZIF-8 (mean particle size 65 nm)	2	25 °C, 4 bar	112.65 (+120%)	108.20	[17]
1657	ZIF-8	5	35 °C, 5 bar	165	44 (CO_2_/N_2_)	[97]
1657	ZIF-8	8	35 °C, 5 bar	175	55 (CO_2_/N_2_)	[98]
1657	ZIF-8	2	30 °C, 1 bar Mixed CO_2_/CH_4_ (30/70 vol%)	102 (Dry) 949 (Humid)	17.3 (Dry) 24.1 (Humid) (CO_2_/CH_4_)	[99]
1657	NH_2_-ZIF-8(10)	6	25 °C, 1 bar	163.8 (+107.6%)	62 (CO_2_/N_2_) (+27%)	[66]
2533/ Pluronic P123 (surfactant)	ZIF-8 (Basolite^®^ Z1200, 1300–1800 m^2^/g; diameter (D50) = 4.9 μm)	5 ZIF-8 2.5 P123	45 °C, 4 bar	328	19.5 (CO_2_/N_2_)	[67]
1657	Zeolitic imidazolate framework cuboid (ZIF-C) nanosheets (thickest ZIF-C nanosheet, 170 nm)	20	25 °C, 2 bar Mixed CO_2_/N_2_ (10/90 vol%) wet (RH = 100%)	387.2	47.1 (CO_2_/N_2_)	[68]
1657	2D imidazole framework hydrophilically modified (hZIF-L) (leaf-like shapes, 5.9 × 2.4 nm)	5	25 °C, 2 bar Mixed gas, wet	502.44	33.82 (CO_2_/CH_4_)	[70]
1657	2-D MIL-96(Al) (150 nm)	25	25 °C, 2 bar Mixed CO_2_/N_2_ (15/85 vol%)	55 (+25%)	67.5 (CO_2_/N_2_) (+18%)	[71]
1657	3-D ZIF-94	25	25 °C, 2 bar Mixed CO_2_/N_2_ (15/85 vol%)	58.5 (+33%)	63 (CO_2_/N_2_)	
1657/ PEG 400	NH_2_-MIL125	12 MOF 40 PEG	25 °C, 2 bar Mixed CO_2_/CH_4_ (10/90 vol%)	190.03 (pure) 183.11 (mixed)	24.84 (pure) 17.05 (mixed) (CO_2_/CH_4_)	[73]
	NH_2_-MIL125	12 MOF 40 PEG	25 °C, 8 bar Mixed CO_2_/CH_4_ (10/90 vol%)	304.76 (pure) 285.45 (mixed)	32.84 (pure) 23.17 (mixed) (CO_2_/CH_4_)	
1657	Bio-ZIF-12	12	25 °C, 2 bar Mixed CO_2_/CH_4_ (20/80 vol%), wet	542	40 (CO_2_/CH_4_)	[75]
1657	Honeycomb-structured UiO-66 (15 nm)	10	20 °C, 3 bar	97.5 (+44.7%)	79.2 (CO_2_/N_2_) 22.1 (CO_2_/CH_4_)	[76]
	Honeycomb-structured amino-functionalized MOF UiO-66-NH_2_ (15 nm)	10	20 °C, 3 bar	118.3 (+49.4%)	56.6 (+71.7%) (CO_2_/N_2_) 30.5 (+34.5%) (CO_2_/CH_4_)	
1657	NOTT-300 (800 nm–1 µm)	40	25 °C, 10 bar	395 (+380%) single 356 (mixed CO_2_/N_2_) 340 (mixed CO_2_/CH_4_)	61.2 CO_2_/N_2_ (+26%) 36.3 CO_2_/CH_4_ (+68%) single 58.36 CO_2_/N_2_ (mixed) 33.24 CO_2_/CH_4_ (mixed)	[74]

^1^ Barrer = 10^−10^ cm^3^ (STP) cm cm^−2^ s^−1^ cmHg^−1^.

**Table 8:** Permeation properties of selected Pebax-based MMMs filled with nanosheets.

Pebax Type	Filler Type	Filler Amount (wt%)	Test Conditions	CO_2_ (Barrer ^1^)	Selectivity (-)	Ref.
1657	MXene (lateral dimension: 1–2 μm; thickness: 1–2 nm)	1	30 °C, 2 bar (dry)	148	63 (CO_2_/N_2_)	[83]
		10	30 °C, 2 bar (humidified)	584	59 (CO_2_/N_2_)	
1657	Layered double hydroxides (LDHs)	2	30 °C, 1 bar Mixed CO_2_/CH_4_ (30/70 vol%)	98.6 (Dry) 619 (Humid)	18.5 (Dry) 28.2 (Humid) (CO_2_/CH_4_)	[99]
1657	Layered double hydroxide nanocage (LDHN)	6	Mixed CO_2_/CH_4_ (10/90 vol%) humidified	426	18	[86]
	Ionic liquid-decorated layered double hydroxide nanocage ([Hmim][NTf_2_]@LDHN)	6	Mixed CO_2_/CH_4_ (10/90 vol%) humidified	644	34 (CO_2_/CH_4_)	
1657	LDHs (lateral dimension 150–200 nm)	2	30 °C, 2 bar Mixed CO_2_/CH_4_ (30/70 vol%)	104 (Dry)	19.1 (Dry) 28 (Humid) (CO_2_/CH_4_)	[87]
				740 (Humid)		
1657	Exfoliation-free laminates’ LDH intercalated with amino acids’ hydrophobic phenylalanine (Phe-LDH) (lateral dimension 100–150 nm)	5		101 (Dry) 760 (Humid)	20.1 (Dry) 36.1 (Humid) (CO_2_/CH_4_)	
1657	Exfoliation-free laminates’ LDH intercalated with amino acids’ hydrophilic glutamic acid (Glu-LDH) (lateral dimension 100–150 nm)	5		109 (Dry) 790 (Humid)	19.8 (Dry) 37.7 (Humid) (CO_2_/CH_4_)	

^1^ Barrer = 10^−10^ cm^3^ (STP) cm cm^−2^ s^−1^ cmHg^−1^.

**Table 9:** Permeation properties of selected Pebax-based MMMs filled with other solid particles.

Pebax Type	Filler Type	Filler Amount (wt%)	Test Conditions	CO_2_ (Barrer ^1^)	Selectivity (-)	Ref.
1657	Nanoadsorbent from oil palm frond (OPF) waste	5	25 °C, 2 bar	1475.09	40.48 (CO_2_/CH_4_)	[95]
1657	Covalent organic frameworks (COFs) COF-5	0.4	30 °C, 1 bar	493	49.3 (CO_2_/N_2_)	[88]
1657	Hollow polypyrrole (PPy) nanospheres	1	35 °C, 2 bar	274	40.1 (CO_2_/N_2_) 12.8 (CO_2_/CH_4_)	[91]
1657/ PEGDME (50/50 wt/wt)	Anion-pillared hybrid ultramicroporous materials GEFSIX-2-Cu-i (Average pore size 3.60 Å; from 200 to 1000 nm)	1	35 °C, 4 bar	460	57 (CO_2_/N_2_) (+9.6%), 18 (CO_2_/CH_4_) (+24.1%) 17 (CO_2_/H_2_) (+12.2%)	[92]
1657/Glycerol	Cu nanoparticles	Gl 15/ Cu 1.5	25 °C, 10 bar	63.6	200	[94]
2533	Amino acid ionic liquids @polymers of intrinsic microporosity (core-shell) composite nanoparticles (AAILs@PIM (core-shell) CNPs) (25–30 nm)	25	65 °C, 2 bar	400	33 (CO_2_/N_2_)	[93]

^1^ Barrer = 10^−10^ cm^3^ (STP) cm cm^−2^ s^−1^ cmHg^−1^.

**Table 10:** Permeation properties of selected Pebax-based MMMs filled with combined fillers.

Pebax Type	Filler Type	Filler Amount (wt%)	Test Conditions	CO_2_ (Barrer ^1^)	Selectivity (-)	Ref.
1657	ZIF-8@CNT	5	35 °C, 5 bar	225.5	48.9 (CO_2_/N_2_)	[97]
1657	MWCNTs@ZIF	8	35 °C, 5 bar	158	49 (CO_2_/N_2_)	[98]
1657	ZIF-8 particles in-situ inserted by multiwalled carbon tubes (MWCNTs@ZIF)	8	35 °C, 5 bar	186.3	61.3 (CO_2_/N_2_)	[98]
1657	Heterostructured filler— in-situ growth of ZIF-8 on LDH surface (ZIF-8@LDH)	2	30 °C, 1 bar Mixed CO_2_/CH_4_ (30/70 vol%)	122 (Dry) 1307 (Humid)	19.2 (Dry) 31.6 (Humid) (CO_2_/CH_4_)	[99]
1657	M-Xene/SiO_2_	0.2/0.8	30 °C, 2 bar	216 (+104%)	61 (CO_2_/N_2_) (+49%)	[84]
1657	M-Xene/HNTs	0.2/0.8		168	51 (CO_2_/N_2_)	
1657	GO/HNTs	0.5/0.5		245 (+153%)	71 (CO_2_/N_2_) (+72%)	

^1^ Barrer = 10^−10^ cm^3^ (STP) cm cm^−2^ s^−1^ cmHg^−1^.

**Table 11:** Permeation properties of selected Pebax-based MMMs filled with ionic and non-ionic additives.

Pebax Type	Filler Type	Filler Amount (wt%)	Test Conditions	CO_2_ (Barrer ^1^)	Selectivity (-)	Ref.
2533	Triglyceride (TPP)	20	35 °C, 5 bar	566	25 (CO_2_/N_2_)	[105]
2533	Tween21	65	25 °C and 0.6 atm	221	32.0 (CO_2_/N_2_)	[100]
	Tween20	65	25 °C and 0.6 atm	267	36.6 (CO_2_/N_2_)	
	Tween80	65	25 °C and 0.6 atm	289	40.70 (CO_2_/N_2_)	
1657	Tween20	50	25 °C, 1 bar	144	50.7 (CO_2_/N_2_) 14.1 (CO_2_/CH_4_)	[101]
	Tween80	50	25 °C, 1 bar	167	47.9 (CO_2_/N_2_) 13.8 (CO_2_/CH_4_)	
1657	Calcium lignosulfonate (CaLS)	Pebax/CaLS(15:1)	25 °C, 3 bar Dry	133	69 (CO_2_/N_2_) 23 (CO_2_/CH_4_)	[103]
		Pebax/CaLS(15:1)	25 °C, 3 bar Humid	3585	29 (CO_2_/CH_4_) 71 (CO_2_/N_2_)	
		Pebax/CaLS(15:1)	85 °C, 3 bar Mixed CO_2_/N_2_ (10/90 vol%) humid	7480	42 (CO_2_/N_2_)	
1657	Aniline	50	25 °C, 7 bar	151 (+76%)	92.5 (CO_2_/N_2_) (+101%)	[7]
		50	25 °C, 7 bar Mixed CO_2_/N_2_ (20/80 vol %)	123.12 (+48%)	68.34 (+262%)	
5513	KBF_4_	0.0045	2 bar	36.8 GPU (CO_2_ permeance)	27.6 (CO_2_/N_2_)	[104]

^1^ Barrer = 10^−10^ cm^3^ (STP) cm cm^−2^ s^−1^ cmHg^−1^.

**Table 12:** Permeation properties of selected Pebax-based thin-film nanocomposite MMMs.

Pebax Type	Filler Type	Filler Amount (wt%)	Test Conditions	CO_2_ Permeance (GPU ^1^)	Selectivity (-)	Ref.
1657 on PAN support with amino-PDMS gutter layer	-	-	20 °C, 5 barg	350	50 (CO_2_/N_2_)	[115]
1657/PEG-DME on PAN support with amino-PDMS gutter layer	-	-	20 °C, 5 barg	400	65 (CO_2_/N_2_)	
1657 on PVDF support	Ionic-Liquid-functionalized graphene oxide (GO-IL)	0.05	25 °C, 4 bar	905 (+50%)	44.8 (CO_2_/N_2_) (+ over 90%) 5.8 (CO_2_/H_2_)	[50]
1657 on P84 support	UiO-66	10	35 °C, 5 bar Mixed CO_2_/CH_4_ (50/50 vol%)	11.5	55.6 (CO_2_/CH_4_)	[64]
1657 on PAN support	MOF-801 nanocrystal 400–500 nm (3 spin coating cycles)	7.5	20 °C, 1 bar Mixed CO_2_/N_2_ (50/50 vol%)	22.4	66 (CO_2_/N_2_)	[77]
1657 on PAN support	2D Mxene Nanosheets	0.15	25 °C, 2 bar	21.6	72.5 (CO_2_/N_2_)	[82]
2533 on polypropylene (PP) hollow fiber supports	UiO-66-NH_2_ (dip coating)	10	25 °C, 2 bar	26	37 (CO_2_/N_2_)	[80]

^1^ GPU = 10^−6^ cm^3^ (STP) cm^−2^ s^−1^ cm Hg^−1^.

## Data Availability

Not applicable.
